# A quantitative and site-specific atlas of the citrullinome reveals widespread existence of citrullination and insights into PADI4 substrates

**DOI:** 10.1038/s41594-024-01214-9

**Published:** 2024-02-06

**Authors:** Alexandra S. Rebak, Ivo A. Hendriks, Jonas D. Elsborg, Sara C. Buch-Larsen, Claus H. Nielsen, Lene Terslev, Rebecca Kirsch, Dres Damgaard, Nadezhda T. Doncheva, Caroline Lennartsson, Martin Rykær, Lars J. Jensen, Maria A. Christophorou, Michael L. Nielsen

**Affiliations:** 1 Proteomics Program, Novo Nordisk Foundation Center for Protein Research, Faculty of Health and Medical Sciences, University of Copenhagen, Copenhagen, Denmark; 2 Institute for Inflammation Research, Center for Rheumatology and Spine Diseases, Rigshospitalet, Copenhagen University Hospital, Copenhagen, Denmark; 3 Copenhagen Center for Arthritis Research, Center for Rheumatology and Spine Diseases, Rigshospitalet, Copenhagen University Hospital, Copenhagen, Denmark; 4 Disease Systems Biology Program, Novo Nordisk Foundation Center for Protein Research, Faculty of Health and Medical Sciences, University of Copenhagen, Copenhagen, Denmark; 5 Epigenetics, Babraham Institute, Cambridge, UK

**Keywords:** Post-translational modifications, Mass spectrometry, Proteomics, Post-translational modifications

## Abstract

Despite the importance of citrullination in physiology and disease, global identification of citrullinated proteins, and the precise targeted sites, has remained challenging. Here we employed quantitative-mass-spectrometry-based proteomics to generate a comprehensive atlas of citrullination sites within the HL60 leukemia cell line following differentiation into neutrophil-like cells. We identified 14,056 citrullination sites within 4,008 proteins and quantified their regulation upon inhibition of the citrullinating enzyme PADI4. With this resource, we provide quantitative and site-specific information on thousands of PADI4 substrates, including signature histone marks and transcriptional regulators. Additionally, using peptide microarrays, we demonstrate the potential clinical relevance of certain identified sites, through distinct reactivities of antibodies contained in synovial fluid from anti-CCP-positive and anti-CCP-negative people with rheumatoid arthritis. Collectively, we describe the human citrullinome at a systems-wide level, provide a resource for understanding citrullination at the mechanistic level and link the identified targeted sites to rheumatoid arthritis.

## Abstract (web summary)

Here the authors leverage state-of-the-art quantitative proteomics to provide a comprehensive overview of the human citrullinome. Supporting evidence from peptide microarrays hints at the potential clinical relevance of some of the identified sites.

## Main

Post-translational modifications (PTMs) are chemical changes that occur on proteins in response to cell stimuli. They are often reversible and act as dynamic molecular switches that modulate protein structure and function, thereby adding enormous complexity to the proteome, and enhance the regulatory potential of cells.

Arginine is converted to citrulline through citrullination, a process that is facilitated by enzymes known as peptidylarginine deiminases (PADIs or PADs). This modification, considered irreversible, involves hydrolyzing the guanidinium group of the arginine side chain, affecting the charge and hydrogen-bonding ability of the amino acid. Humans have five PADI isoforms (PADI1–PADI6), each of which has different tissue and cellular distributions^1,2^. Citrullination can affect interactions with nucleic acids and proteins, potentially altering protein structures^3–5^.

Citrullination has functional roles in various biological processes^5–9^. Evidence suggests that it is involved in various disorders, including autoimmunity, neurodegeneration, atherosclerosis and cancer^10–12^.

Citrullination in neutrophils is attracting increasing attention as a mediator of rheumatoid arthritis (RA)^13,14^ and systemic lupus erythematosus (SLE)^15,16^. In RA, increased protein citrullination in inflamed joints, the presence of autoantibodies (ACPAs) and the genetic association of PADI4 with RA development underscore the importance of this process^17–19^. ACPAs, detected through anti-cyclic citrullinate peptide/protein (CCP) assays, are diagnostic and prognostic markers of RA^20^. Inhibition of PADI activity has shown promise in disease models, making it a potential therapeutic strategy for RA, ulcerative colitis, neurodegeneration and cancer^2,21^.

Despite great biological and clinical interest, the understanding of citrullination and its associated molecular mechanisms remains limited. Mapping citrullinated proteins and modification sites has been challenging and limited by contemporary analytical strategies. Robustly sensitive enrichment methods are lacking, and mass spectrometers are unable to discern specific, low-abundance citrullination events in complex cell extracts^22–27^.

To comprehensively map citrullination with high precision, we used advanced proteomics technology. Our approach involved offline high-pH reversed-phase fractionation^28^ followed by online low-pH reversed-phase fractionation, coupled with high-resolution mass spectrometry (MS). We delineated the human citrullinome and identified specific citrullination sites catalyzed by PADI4. To capture the functional and biological aspects of the process, we used HL60 promyeloblasts as a model for human neutrophil behavior. Following DMSO treatment, these cells differentiated into neutrophil-like cells (NLCs), increasing PADI4 enzyme expression^29^. We induced PADI enzyme activity by elevating cellular calcium ion concentrations and assessed citrullination catalysis through quantitative MS.

Collectively, our analyses identified 14,056 high-confidence citrullination sites on 4,008 proteins, vastly expanding the number of known sites by >16-fold. Identified citrullination events occurred on core histone variants and non-histone proteins, broadening our understanding of citrullination’s regulatory functions and underscoring the current underestimation of citrullinated autoantigens in neutrophils. Using a peptide microarray, we investigated ACPA specificity in synovial fluid from people with RA or ankylosing spondylitis, revealing differences in reactivity and binding motifs. Collectively, our findings demonstrate the extensive regulatory role of citrullination, showcasing how MS data can enhance our understanding of autoantigens.

## Results

**Fig. 1:**
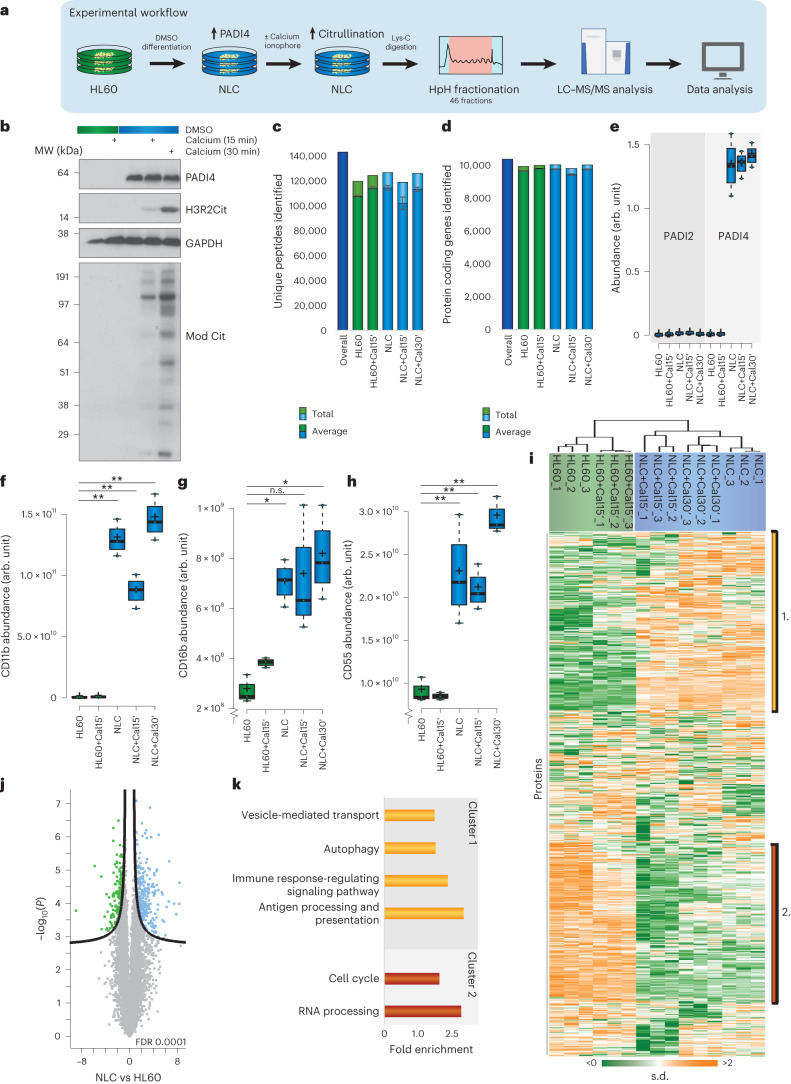
Establishment of regulatory citrullination cell system. **a**, Schematic of the workflow. HL60 cells were cultured in standard RPMI medium or differentiated by DMSO into neutrophil-like cells (NLCs). Cells were then incubated in Locke’s solution with 4 µM calcium ionophore for 0, 15 or 30 min. Following Lys-C digestion, the peptides were separated by high-pH (HpH) fractionation and analyzed using high-resolution LC–MS/MS. Each condition was prepared in triplicate. **b**, Western blot analysis of PADI4, H3R2Cit and modified citrulline shows that PADI4 is expressed upon DMSO differentiation and activated following calcium treatment. The experiment was repeated three times independently, with similar results. The modified citrulline was run on a different gel than was the GAPDH loading control, owing to different sample-preparation requirements. MW, molecular weight. **c**, The number of cumulative peptides detected and across the conditions. *n* = 3 biologically independent samples. Data are presented as mean values ± s.d. Cal15' and Cal30', calcium treatment for 15 or 30 min, respectively. **d**, The number of cumulative proteins detected and across the conditions. *n* = 3 biologically independent samples. Data are presented as mean values ± s.d. **e**, Abundance of PADI2 and PADI4 across conditions, based on label-free quantification (LFQ) intensity. *n* = 3 biologically independent samples; whiskers, 95th and 5th percentiles; box limits, third and first quartiles; center bar, median; ‘+’ symbol, average. arb. unit, arbitrary unit. **f**, As in **e**, but for CD11b. **g**, As in **e**, but for CD16b. **h**, As in **e**, but for CD55. **i**, Hierarchical clustering analysis of *z*-scored intensity values of all proteins detected across all conditions. Orange, upregulated; green, downregulated. Bars 1. and 2. correspond to clusters 1 and 2, respectively, as analyzed in **k**. **j**, Volcano plot for visualization of protein dynamics in response to DMSO differentiation, from the HL60 to NLC condition. FDR, 0.0001. **k**, GO term enrichment analysis for biological processes of citrullination target proteins of selections displayed in **d**, as compared with the detected HL60 proteome. Statistical information is available in Supplementary Table 5. For **f**–**h**, significance was determined by two-tailed Student’s *t* test with no correction for multiple comparisons. Asterisks indicate significant difference between HL60 and the indicated conditions: *P < 0.05; **P < 0.001; n.s., non-significant. Source data

To identify citrullinated proteins and their modification sites globally, we used a proteomics-based approach with human promyelocytic leukemia cells (HL60) with or without calcium ionophore. Recognizing the challenge of detecting low-abundance citrullination sites in complex samples^30^, we implemented offline high-pH reversed-phase fractionation into 46 fractions to reduce sample complexity^28^ (Fig. 1a). This strategy, combined with the high sequencing speed of modern MS instrumentation^31^, enabled direct analysis of citrullination events, without needing PTM-specific enrichment.

As a first step, we confirmed by western blot that DMSO had induced differentiation of HL60 cells into NLCs. The blot revealed that the PADI4 enzyme was upregulated (Fig. 1b). The addition of a calcium ionophore enhances the catalytic activity of PADI4. Because prolonged treatment can recapitulate neutrophilic extracellular trap (NET) formation in vitro^32^, we opted for 15- and 30-min treatments, which resulted in a total of five biological conditions: (1) HL60, no calcium; (2) HL60, 15 min calcium; (3) NLCs, no calcium; (4) NLCs, 15 min calcium; and (5) NLCs, 30 min calcium. The western blot detected profound global citrullination, including citrullinated histone H3R2 (H3R2Cit)^33^, a known citrullination target, as well as modified citrulline, detected by a pan-peptidyl-citrulline antibody (Mod Cit) (Fig. 1b). For improved identification and localization of citrullination sites, samples were digested with endoproteinase Lys-C, ensuring that the arginine residues targeted for citrullination were positioned internally in the analyzed peptide sequences. Each sample fraction was analyzed on short gradients using high-resolution MS (Q-Exactive HF-X)^34^. This allowed the citrullinome to be measured in less than 3 d. Quicker analysis could be achieved by analyzing fewer fractions. In total, we analyzed 138 liquid chromatography–tandem MS (LC–MS/MS) runs for each condition, and performed rigorous and stringent data filtering using MaxQuant to ensure that identification and quantification of citrullinated peptides was robust, with a high degree of confidence.

### Proteome changes upon PADI activation

**Extended Data Fig. 1:**
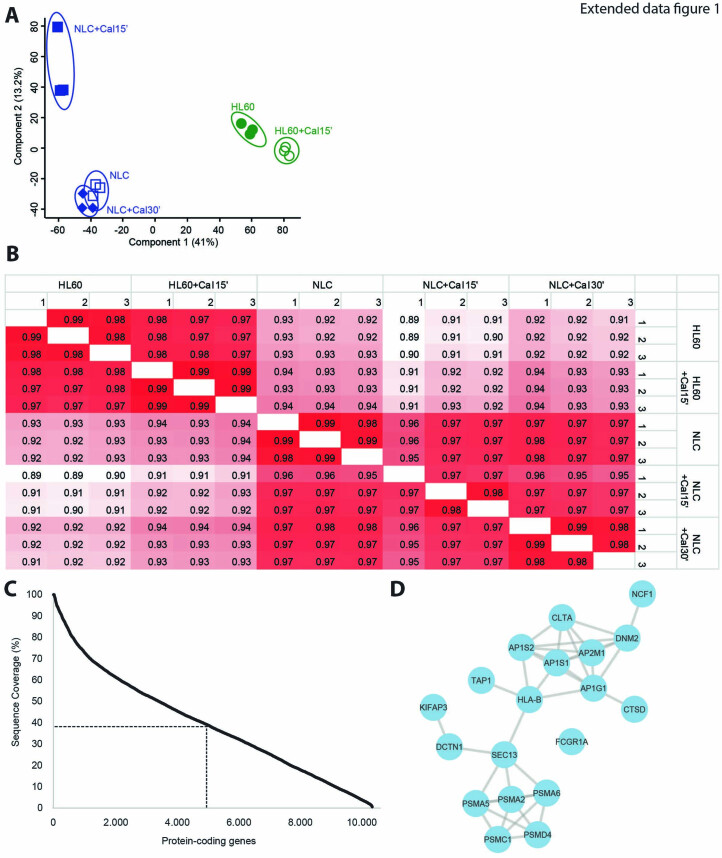
Validation of experimental system. **a**) Principal component analysis of all conditions at protein level. Eigenvalues are displayed on the axes. **b**) Pearson correlation of proteins detected in all replicates of experimental conditions. Clear distinction detection in proteome following DMSO-differentiation. **c**) Sequence coverage of proteins detected. Dotted line indicates median. **d**) STRING network visualizing functional associations between proteins annotated to be involved in antigen processing and presentation detected as upregulated in NLCs (Cluster 1, Fig. 1i, k). Default STRING confidence score cutoff of > 0.4 was used.

Because we performed high-pH fractionation on whole-cell lysate and did not perform a PTM-specific enrichment step^28^, our proteomics strategy allows for investigation of cellular protein-expression changes (that is, the proteome) concomitantly with the profiling of the citrullinome^28^. Overall, the biological replicates of the proteome measurements demonstrated high quantification accuracy and reproducibility, with a clear distinction between HL60 and NLC cells (Extended Data Fig. 1a) with Pearson correlation coefficients of 0.97–0.99 between replicate analyses (Extended Data Fig. 1b). We identified a total of 143,403 unique peptides, with a false discovery rate (FDR) of 1% across experimental conditions; in each individual condition, >100,000 unique peptides were identified (Fig. 1c and Supplementary Table 1). The identified peptides mapped onto a total of 10,630 unique protein-coding genes, and more than 9,400 unique protein-coding genes were quantified across individual experiments (Fig. 1d and Supplementary Table 2). The capacity of high-pH fractionation to improve peptide sequence coverage of identified proteins has been demonstrated^28^. Likewise, our analysis yields a median sequence coverage of 37% for the identified proteins (Extended Data Fig. 1c).

Consistent with our observations using western blotting (Fig. 1b) and literature reports^29^, our proteome analysis confirms that expression of PADI4 is significantly induced upon the differentiation of HL60 cells into NLCs. These observations align with high expression levels of PADI4 in neutrophils^35^. Notably, the increased expression of PADI4 occurred before cells were treated with calcium ionophore, confirming that PADI4 upregulation was not merely an indirect consequence of ionophore stimulation (Fig. 1e). Neutrophils are known to express PADI4 and PADI2 (refs. ^35,36^). We found evidence for low expression of PADI2, but no changes in expression were observed across the investigated cellular conditions (Fig. 1e). Despite our deep proteome analysis, we found no evidence for expression of PADI enzymes besides PADI4 and PADI2 (Supplementary Table 2). We conclude that PADI2 and PADI4 are likely the enzymes that are responsible for citrullination catalysis in the analyzed cells. Expression levels of previously reported neutrophil markers, including CD11b (Fig. 1f), CD16b (Fig. 1g) and CD55 (Fig. 1h)^37,38^, were increased in expression upon differentiation into NLCs.

Our protein-expression data enable a systems-level assessment of the effects of DMSO treatment on the proteome. Our data set provides a foundation for understanding the global protein-expression patterns underlying functional specialization of HL60 cells into NLCs. To obtain a functional classification of the differences related to differentiation, we performed unsupervised hierarchical clustering of the >10,000 identified proteins (Fig. 1i). The resulting heat map revealed one major cluster of proteins that is highly expressed in differentiated NLCs (Fig. 1i,j), with Gene Ontology (GO) analysis revealing enrichment (*P* < 0.006) of terms related to neutrophil functions, including immune-response-regulating pathways, autophagy, antigen processing and presentation, and vesicle-mediated transport (Fig. 1k and Extended Data Fig. 1d). Contrary to this, a second major cluster (cluster 2) comprised proteins with high expression in undifferentiated HL60 cells; it was enriched in biological processes such as cell cycle and RNA processing. In summary, our results affirm the differentiation of HL60 cells into NLCs, establishing relevant biological conditions conducive for studying citrullination events mediated by the PADI4 enzyme.

### Proteome-wide identification of citrullination sites

**Fig. 2:**
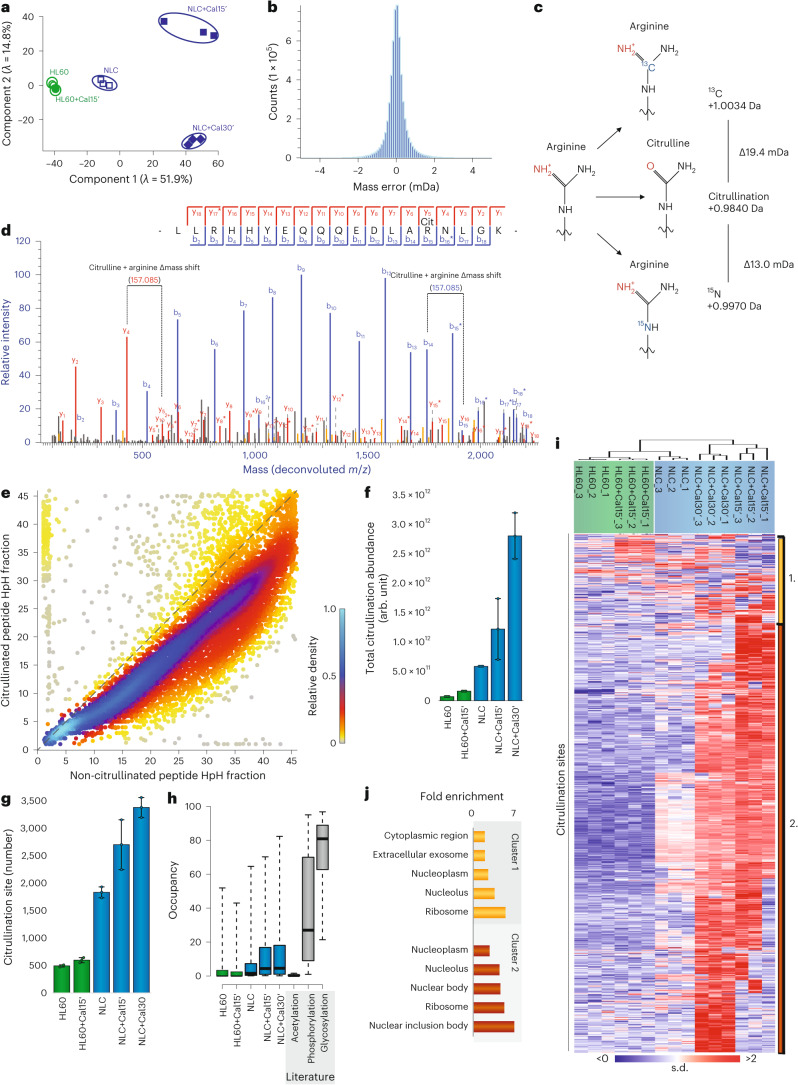
Validation of regulatory citrullination cell system. **a**, PCA of all conditions at the citrullination-site level. Eigenvalues are displayed on the axes. **b**, Mass accuracy of all identified peptides. **c**, Diagram displaying small mass shifts between citrullination, carbon-13 (^13^C) and nitrogen-15 (^15^N) substitution on residue. **d**, Representative MS/MS spectrum, showing localized citrulline. b ions (blue) and y ions (red) are shown; neutral loss is indicated with an asterisk. **e**, Density plot of the fraction distribution during high-pH reversed-phase separation for unmodified peptides (*x* axis) compared with citrullinated peptides (*y* axis). **f**, Total citrullination abundance of sites detected across conditions. *n* = 3 biologically independent samples. Data are presented as mean values ± s.d. **g**, Number of citrullination sites detected in the five conditions by MS/MS and matching. Localization score > 0.75. *n* = 3 biological independent samples. Data are presented as mean values ± s.d. **h**, Occupancy of citrullination across the five conditions. For comparison, occupancies of acetylation^90^, phosphorylation^91^ and glycosylation^119^ are shown. Whiskers, 95th and 5th percentiles; box limits, third and first quartiles; center bar, median; ‘+’ symbol, average. **i**, Hierarchical clustering analysis of *z*-scored intensity values of all proteins detected across all conditions. Red, upregulated; blue, downregulated. **j**, Gene Ontology term enrichment analysis for biological process of citrullination target proteins in HL60 cells and the NLC+Cal30' condition, in addition to selections displayed in **i** (clusters 1 and 2), as compared with the human proteome. Statistical information is available in Supplementary Table 5. Source data

**Extended Data Fig. 2:**
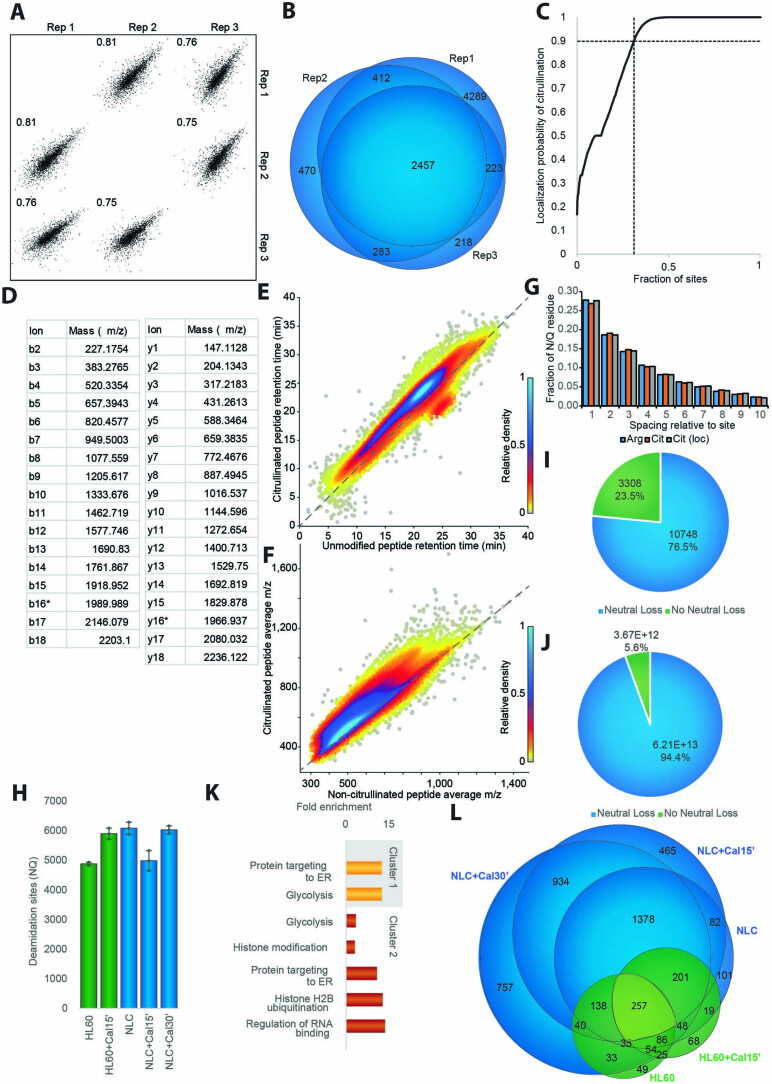
Validation of detection of citrullination sites. **a**) Pearson correlation of citrullination sites detected in all three replicates of NLC+Cal30’ condition. **b**) Venn diagram showing site detection overlap between all three replicates of NLC+Cal30’ condition. **c**) Citrullination localization probability plotted against the ranked fraction of all peptide-spectrum-matches (PSMs). Despite all probabilities being displayed, only those above 0.9 were used for assignment of unique citrullinated peptides and sites. **d**) Mass of b and y ions shown in MS/MS spectrum in Fig. 2c. **e**) Density plot of elution time during low pH reversed-phase separation for unmodified peptides (x-axis) vs citrullinated peptides (y-axis). **f**) Density plot of detected m/z for unmodified peptides (x-axis) vs m/z for citrullinated peptides (y-axis). **g**) Fraction of N/Q residues relatives to citrullination site spacing for unmodified arginine residues, citrullination sites and localized citrullination sites. **h**) Number of deamidation sites of asparagine (N) and glutamine (Q) detected across conditions. n = 3 biological independent samples,. Data are presented as mean values ± SD. **i**) Pie chart showing distribution of sites detected with neutral loss of cyanic acid or no neutral loss. **j**) Pie chart showing citrullination abundance associated with sites for which a neutral loss was either detected or not detected. **k**) Gene Ontology (GO) term enrichment analysis for cellular components of citrullination target proteins in selections displayed in 1 G (cluster 1 and 2), as compared to the human proteome. Statistical information in Supplementary Table 5. **l**) Scaled venn diagram displaying overall of number sites detected in each experimental condition.

Having established that NLCs express PADI4, we next evaluated the capability of our methodology to identify citrullination sites. Overall, we observed a high degree of reproducibility across biological replicates of NLCs (Extended Data Fig. 2a), which resulted in ~70% overlap in citrullination sites between any two replicate runs of NLCs (Extended Data Fig. 2b). For all investigated cells and treatments, a principal component analysis (PCA) analysis revealed clear separation between the individual cell populations (Fig. 2a). Reassuringly, the high precursor-mass accuracy of the MS analysis (<2 mDa) ensured that the mass increment caused by citrullination (~0.9840 Da) could be easily distinguished from the change in peptide mass resulting from the naturally occurring stable isotopes of carbon and nitrogen (^13^C, +1.0034 Da; ^15^N, +0.9970 Da)^39^ (Fig. 2b,c).

To determine the location of citrullination sites within the identified peptides, we used high-resolution MS/MS analysis. Each identified spectrum was assigned a localization score to indicate the confidence level associated with the identification of the amino acid harboring the citrullination modification (Fig. 2d and Extended Data Fig. 2d). Because citrullination of arginine residues results in the same peptide mass shift as deamidation of glutamine and asparagine residues, we considered only citrullination sites identified from MS/MS spectra with high localization probability (>0.9) (Extended Data Fig. 2c), thereby ensuring high confidence in distinguishing between amino acids that become citrullinated or deamidated. The average localization probability of all identified citrullination sites in our filtered data set was 99.41%.

To further confirm our ability to differentiate citrullination from deamidation, we investigated the chromatographic elution times of citrullinated peptides and compared these with those of unmodified peptides and deamidated peptides. We found that there was distinct chromatographic separation between citrullinated peptides and their unmodified counterparts (Fig. 2e and Extended Data Fig. 2e), that citrullinated peptides and deamidated peptides had different *m/z* ratios (Extended Data Fig. 2f) and that there was no spatial bias of citrullination site location relative to asparagine (N) and glutamine (Q) targets for deamidation (Extended Data Fig. 2g), confirming robust differentiation between citrullination and deamidation events (see Supplementary Note 1 for further details).

Our pilot analysis resulted in high-confidence identification of 5,238 citrullination sites across the investigated cell conditions (Supplementary Table 3). Within these data, we investigated which proteins were most abundantly modified with citrullination, regardless of the total number of modification sites, encompassing proteins associated with various biological processes (Table 1). The highest number of citrullination sites was identified in NLCs after 30-min stimulation with calcium ionophore (Fig. 2g), in agreement with western blot observations (Fig. 1b). Reassuringly, we observed that >75% of the acquired citrullinated MS/MS spectra (Extended Data Fig. 2i) exhibited a consistent neutral loss of isocyanic acid, representing ~95% of the total citrullinated peptide abundance (Extended Data Fig. 2j). This distinctive characteristic serves as a reliable signature for the confident identification of citrullination sites^40^. In contrast to the increased citrullination abundance in NLCs (Fig. 2f), we observed no significant change in the number of deamidation sites (Extended Data Fig. 2h) across experimental conditions. This observation underscores that our high-resolution proteomics data allows citrullination to be distinguished from deamidation. We found that the increase in citrullination-site abundance between 15 and 30 min of calcium ionophore treatment (Fig. 2f) was higher than the corresponding increase in the number of citrullination sites for the same conditions (Fig. 2g). This indicates that PADI4 activation is specific and tightly regulated, with longer periods of calcium ionophore stimulation leading to enhanced citrullination of the same arginine residues.

**Table 1:** Top 50 most abundantly citrullinated proteins identified in system validation study on the HF-Exactive system

Gene name	Total number of sites	Most abundant site	Second most abundant site	Third most abundant site	Biological process
*H3C1*	1	R27	–	–	Chromatin organization
*ALYREF*	3	R141	R144	R253	mRNA processing
*MNDA*	3	R127	R119	R129	Activation of innate immune response
*RPL24*	9	R92	R140	R88	mRNA processing
*H3-3A*	6	R9	R27	R18	Telomere organization
*RSL24D1*	10	R157	R80	R19	Translational initiation
*NIPSNAP3B*	2	R96	R100	–	Mitophagy regulation
*ENO1*	6	R400	R403	R50	Canonical glycolysis
*H1-4*	2	R54	R33	–	Chromatin organization
*H2AC4*	3	R43	R4	R72	Chromatin organization
*HNRNPA3*	9	R113	R377	R376	mRNA processing
*H1-10*	6	R128	R127	R155	Chromatin organization
*SUB1*	2	R47	R59	–	Negative regulation of DNA metabolic process
*HMGB1*	4	R24	R73	R70	Activation of innate immune response
*HNRNPA2B1*	11	R352	R21	R350	mRNA processing
*NPM1*	6	R197	R142	R277	Cell aging
*RPL29*	7	R95	R18	R68	Translational initiation
*SERF2*	7	R26	R7	R3	Protein destabilization
*CORO1A*	7	R3	R12	R402	Actin filament organization
*TAGLN2*	6	R12	R4	R160	Platelet degranulation
*HNRNPM*	5	R56	R60	R707	mRNA processing
*PTMA*	1	R90	–	–	Transcription
*RPL10*	8	R24	R203	R21	Translational initiation
*RPS10*	7	R124	R153	R119	Translational initiation
*DEK*	10	R75	R8	R65	Chromatin organization
*MSN*	6	R503	R408	R393	Cellular response to testosterone stimulus
*SRSF3*	1	R3	–	–	Cellular response to leukemia inhibitory factor
*LBR*	9	R141	R96	R95	Neutrophil differentiation
*HNRNPU*	12	R255	R268	R250	mRNA processing
*ARPC1B*	4	R294	R299	R167	Response to estrogen
*RPL31*	3	R14	R92	R23	Translational initiation
*DNAJC8*	8	R224	R216	R190	mRNA processing
*LCP1*	5	R3	R530	R141	Actin filament organization
*CBX3*	3	R108	R91	R171	Chromatin organization
*NOP58*	3	R401	R404	R443	rRNA processing
*KHDRBS1*	2	R442	R436	–	mRNA processing
*THRAP3*	17	R595	R513	R886	mRNA processing
*HCLS1*	11	R93	R155	R58	Actin filament polymerization
*RPL17*	6	R18	R69	R23	Translational initiation
*NCL*	6	R567	R390	R561	Angiogenesis
*PCNP*	3	R137	R164	R47	Cell cycle
*H1*-5	1	R57	–	–	Chromatin organization
*EDF1*	5	R63	R34	R33	Endothelial cell differentiation
*XRCC5*	4	R178	R368	R640	Activation of innate immune response
*DDX3X*	7	R121	R46	R75	Cell differentiation
*RPL32*	6	R27	R130	R22	Translational initiation
*FAM107B*	3	R43	R35	R28	Alternative splicing
*RPL19*	16	R16	R38	R117	Translational initiation
*PPIA*	6	R55	R148	R144	Protein folding
*HSP90AA1*	7	R620	R647	R464	Axon extension

Next, we wanted to assess the ‘citrullination modification stoichiometry’ (that is, the modification percentage of any given arginine residue, also referred to as occupancy), because such information is valuable for understanding the implications of PTMs in protein regulation. Although stoichiometry in itself does not provide direct evidence of biologically relevant function, it is expected that sites with higher modification stoichiometries are more likely to have functional consequences^41^. Across the citrullinome, we observed that the modification stoichiometry increased from ~7% for the HL60 cells to ~16% for the NLC cells upon DMSO-induced differentiation and 30-min calcium ionophore treatment (Fig. 2h). We compared citrullination stoichiometry with the previously published stoichiometry of other PTMs, and found that it was larger than the stoichiometry of acetylation yet lower than the overall stoichiometry of phosphorylation and N-glycosylation (Fig. 2h).

### Cellular and functional classification of targeted proteins

To obtain an overview of the subcellular compartments and cellular functions that citrullinated proteins associate with, we first clustered the identified citrullination sites into a heat map, which revealed two main clusters (Fig. 2i): one centered on baseline modification sites (cluster 1; yellow), and another containing the majority of citrullination sites upon cell stimulation (cluster 2; red). Next, we performed GO term enrichment analysis to extract the over-represented categories in the identified citrullination clusters as compared with the human proteome. GO Cellular Component (GOCC) analysis highlighted that citrullination sites induced upon differentiation into NLCs and subsequent induction of PADI generally localized to the nucleus, particularly to the nucleolus, nucleoplasm and nuclear inclusion bodies (Fig. 2j). Modification of the ribosome was also observed, in alignment with previous observations^7,42^. The observed nuclear localization of citrullinated proteins aligns with the reported cellular localization of PADI4. Citrullination sites that remained unchanged after cellular perturbation (cluster 1) were predominantly associated with proteins located in the cytosol and extracellular exosome. These sites seemed to be catalyzed by baseline PADI enzyme activity, which remained unaffected by the cellular treatments in this study. GO Biological Processes (GOBP) analysis of the two clusters (Extended Data Fig. 2k) indicated an enrichment for protein targeting to ER and glycolysis in cluster 1, whereas cluster 2 demonstrated an enrichment for histone modification and proteins involved in regulation of RNA binding.

Notably, we identified ~500 citrullination sites in HL60 cells (Fig. 2g) that were not visible in western blot analysis (Fig. 1b). This observation suggests the presence of low-level activation of PADI enzymes and underscores the enhanced sensitivity of our proteomic approach when compared to Western blotting. Among these citrullination sites, 257 were consistently identified across all five experimental conditions (Extended Data Fig. 2l), supporting the hypothesis that they might constitute a basal ‘core citrullinome’. The proteins encompassed within this core citrullinome displayed a significant enrichment in functions related to translation and RNA processing, which suggests that these central cellular functions may be regulated by citrullination under unperturbed cellular conditions.

### Characterizing the PADI4-specific citrullinome

**Fig. 3:**
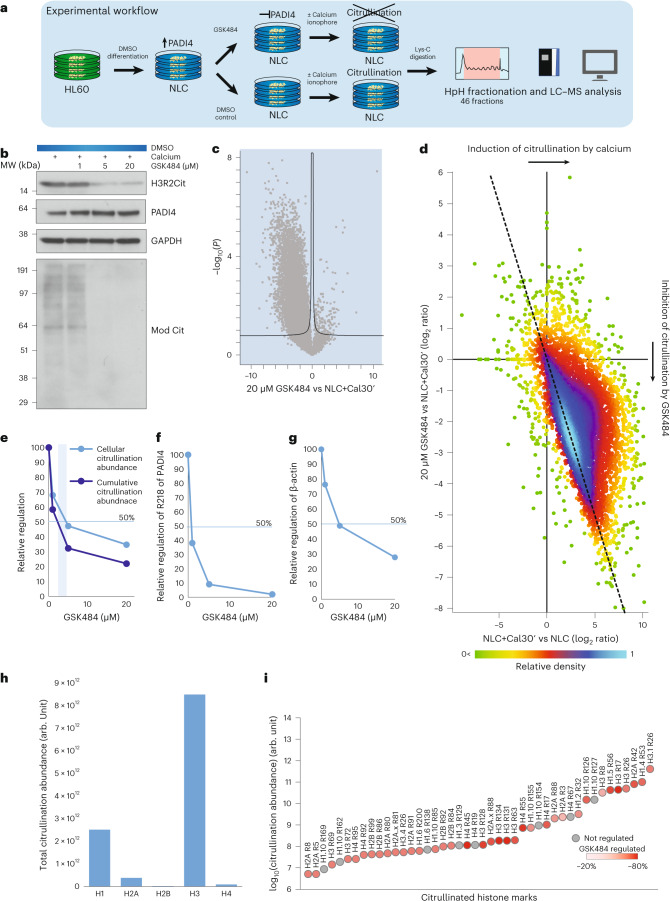
PADI4 inhibition by GSK484. **a**, Schematic representation of the workflow. HL60 cells were cultured in standard RPMI medium and differentiated by DMSO to neutrophil-like cells (NLCs). Cells were then incubated in Locke’s solution, with varying concentrations (1 µM, 5 µM, 20 µM) of the PADI4 inhibitor GSK484 or a control for 1 h prior to treatment with 4 µM calcium ionophore for 30 min. Following Lys-C digestion, the peptides were separated by high-pH fractionation and analyzed using high-resolution LC–MS/MS. Each condition was prepared in quadruplicate. **b**, Western blot analysis of PADI4, H3R2Cit and modified citrulline shows that PADI4 activation by calcium treatment is increasingly inhibited by GSK484. The experiment was repeated three times independently, with similar results. The modified citrulline was run on a different gel than was the GAPDH loading control owing to different sample-preparation requirements. **c**, Volcano plot displaying regulation of sites in response to treatment with 20 µM GSK484. **d**, Density plot of sites detected in response to calcium activation (across *x* axis) and 20 µM GSK484 treatment (*y* axis). The dashed line is equivalent to *y* = –*x*. **e**, Regulation of total citrullination abundance and significantly regulated sites in response to increasing GSK484 concentration. The vertical bar represents the concentration range of GSK484 to achieve 50% downregulation of citrullination abundance between total citrullination abundance and the significantly regulated sites. **f**, Regulation of the autocitrullination site R218 of PADI4 in response to increasing GSK484 concentrations relative to control. **g**, Regulation of citrullination on β-actin in response to increasing GSK484 concentrations relative to control. **h**, Total citrullination abundance for histone variants. **i**, Diagram showing citrullination abundance of citrullinated histone marks and their level of regulation in response to 20 µM GSK484. Source data

**Extended Data Fig. 3:**
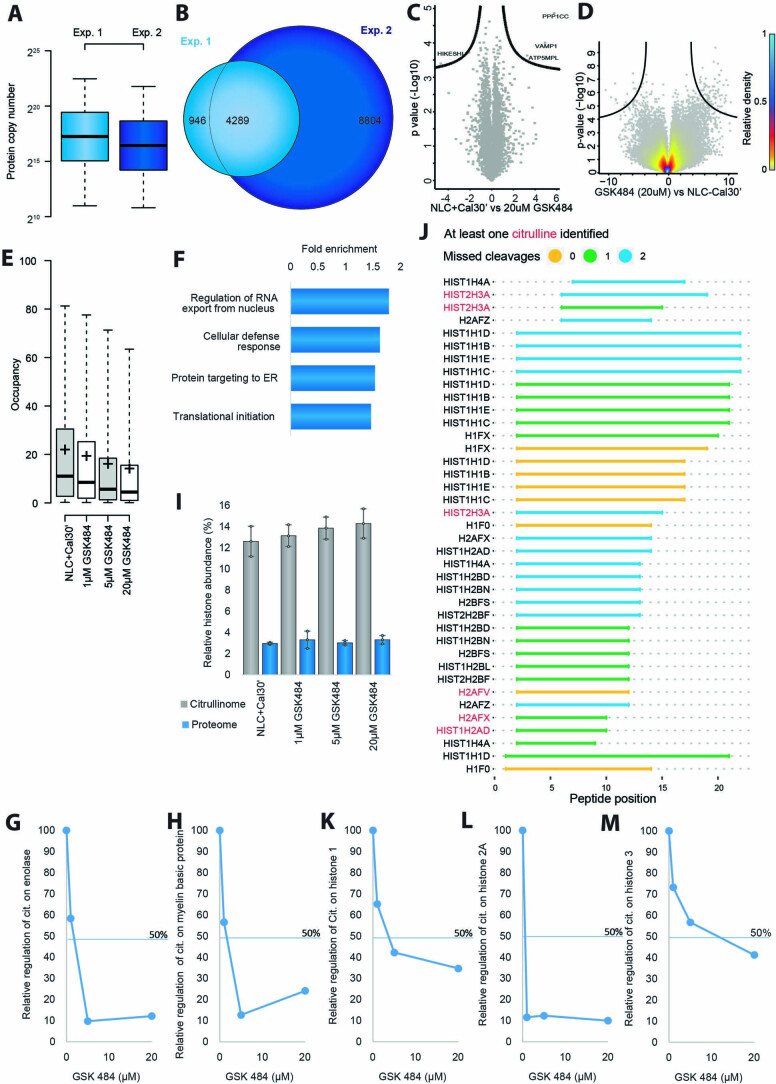
Regulation of PADI4 by specific inhibitor, GSK484. **a**) Protein copy number of proteins detected in pilot study (Exp. 1) (Figs. 1 and 2) and GSK experiment (Exp. 2) with optimized deeper sequencing. Whiskers: 95th and 5th percentile, box limits; 3rd and 1st quartiles, center bar; median. n = 3 biological independent samples for Exp. 1 and n = 4 biological independent samples for Exp. 2. **b**) Venn diagram displaying distribution of sites detected in pilot study (Figs. 1 and 2) performed on HF-Exactive and optimized GSK experiment performed on Exploris systems. **c**) Volcano plot of protein detected displaying little significant protein regulation in response to treatment with 20 µM GSK484. **d**) Volcano plot of unmodified peptides from proteins displaying significant regulation of citrullination detected during treatment with 20 µM GSK484 vs control condition. **e**) Occupancy of citrullination across the four conditions. Whiskers: 95th and 5th percentile, box limits; 3rd and 1st quartiles, center bar; median, +symbol; average. **f**) Gene Ontology (GO) term enrichment analysis for biological processes of citrullination targeted proteins in the 20 µM GSK484 condition, as compared control condition without inhibitor. **g**) Regulation of citrullination on enolase in response to increasing GSK484 concentrations relative to control. **h**) Regulation of citrullination on myelin basic protein in response to increasing GSK484 concentrations relative to control. **i**) Relative histone abundance in citrullinome (grey) and proteome (blue). n = 4 biological independent samples, error bars represent SD. **j**) Histone N-terminal coverage by histone isoform. Citrullination site in terminal indicated (red). Missed cleavage indicated; 0 (yellow), 1 (green) and 2 (blue). **k**) Regulation of citrullination on histone 1 variant in response to increasing GSK484 concentrations relative to control. **l**) Regulation of citrullination on histone 2A in response to increasing GSK484 concentrations relative to control. **m**) Regulation of citrullination on histone 3 in response to increasing GSK484 concentrations relative to control. Source data

We next aimed to identify substrates specifically citrullinated by PADI4, by performing quantitative citrullination MS analysis of cells treated with the PADI4-specific inhibitor GSK484 (ref. ^43^) (Fig. 3a). To this end, we treated NLCs with GSK484 for 30 min before the calcium-induced PADI4 activation, and compared GSK484-treated cells with their mock-treated counterparts. All experiments were performed in quadruplicate. To elucidate the concentration-dependent effect of the inhibitor, we performed an analysis across three inhibitor concentrations (1 µM, 5 µM and 20 µM); strong differences in citrullination were observed in western blot results (Fig. 3b). For the MS analysis, we switched to the Orbitrap Exploris 480 mass spectrometer^44^, which improved the sensitivity of the MS methodology. As a result, we observed improved sequencing depth in the analyzed samples (Extended Data Fig. 3a), which overall led to a deeper coverage of the citrullinome (Extended Data Fig. 3b) and improved identification of a total of 14,056 citrullination sites (Supplementary Table 3) on 4,008 protein-coding genes (Supplementary Table 4).

Overall, no changes in protein-expression level were observed when cells were treated with GSK484 (Extended Data Fig. 3c). We defined significantly regulated citrullination sites on the basis of both significance and fold change (shown in the volcano plot in Fig. 3c). No global significant reduction in the non-modified peptides from the same target proteins was observed (Extended Data Fig. 3d). Overall, treating cells with GSK484 led to a global reduction in citrullination signal (Fig. 3c) and to a concomitant decrease in citrullination-site stoichiometry proportionally with increasing concentrations of GSK484 (Extended Data Fig. 3e). To investigate whether the citrullination sites induced via calcium activation of PADI4 correlated with the ones inhibited by GSK484, we compared the increase of citrullination observed upon calcium treatment with the corresponding site-specific changes detected upon inhibition with 20 µM GSK484 (Fig. 3d). The strong negative correlation between the two data sets confirmed that the majority of citrullination sites induced upon calcium stimulation of NLCs are PADI4 targets. From the volcano plot (Fig. 3c), we found that 4,957 citrullination sites, located on >2,000 target proteins of citrullination mediated by PADI4, were significantly downregulated upon treatment with GSK484. This corresponded to more than 50% of all identified citrullination target proteins, demonstrating that the regulatory scope of PADI4 is much larger than previously thought^27,30^. Next, we assessed the biological processes associated with PADI4-specific citrullination sites and performed GO analysis. From this, we identified an enrichment for regulation of RNA export from the nucleus and translational initiation, which matches the core citrullinome (Extended Data Fig. 3f). Collectively, we identified citrullination sites on many previously reported ACPAs (Supplementary Table 3), including 44 specifically related to RA, on the basis of the AAgAtlas^45^ (Table 2).

**Table 2:** Citrullinated RA autoantigens found in proteomics data set

Gene name	No. of sites	Protein (Uniprot ID)	Citrullination positions
*LMNB1*	19	P20700	R10; R14; R51; R67; R136; R197; R220; R221; R226; R234; R250; R258; R315; R336; R397; R402; R407; R410; R413
*LMNA*	17	P02545	R7; R8; R11; R216; R220; R225; R235; R240; R275; R280; R386; R388; R397; R399; R401; R419; R427; R571;R572
*HNRNPA2B1*	12	P22626	R12; R21; R54; R82; R95; R99; R147; R153; R185; R325; R350; R352
*DEK*	11	P35659	R29; R58; R65; R75; R93; R107; R116; R153; R168; R208; R215
*HNRNPA1*	10	P09651	R31; R47; R75; R88; R92; R97; R122; R140; R146; R178
*GTF2F1*	10	P35269	R46; R49; R73; R101; R180; R181; R206; R413; R422; R424
*TOP1*	9	P11387	R17; R138; R140; R163; R210; R362; R364; R693; R708
*ENO1*	9	P06733	R32; R50; R56; R132; R253; R372; R400; R403; R412
*HSPA5*	9	P11021	R49; R60; R74; R197; R336; R532; R540; R558; R562
*PARP1*	8	P09874	R10; R156; R208; R282; R340; R355; R496; R806
*MBP*	7	P02686	R23; R37; R88; R93; R97; R102; R110
*CAST*	7	P20810	R86; R206; R269; R301; R310; R378; R610
*HMGB2*	6	P26583	R10; R24; R70; R73; R110; R177
*VIM*	6	P08670	R113; R145; R304; R310; R424; R450
*PTPN22*	5	Q9Y2R2	R357; R458; R510; R672; R756
*HMGB1*	5	P09429	R70; R73; R110; R163; R24
*RAF1*	5	P04049	R73; R316; R318; R391; R627
*PADI4*	4	Q9UM07	R218; R372; R374; R394
*HSP90AB1*	4	P08238	R291; R506; R604; R612
*CAT*	4	P04040	R5; R263; R458; R522
*SSB*	4	P05455	R90; R297; R384; R386
*TKT*	3	P29401	R205;R471;R474
*HMGN2*	3	P05204	R23; R24; R27
*HNRNPD*	3	Q14103	R272; R280; R282
*CENPB*	3	P07199	R66; R588; R593
*YBX1*	3	P67809	R69; R77; R101
*ANXA1*	3	P04083	R72; R144; R298
*ANXA6*	2	P08133	R11; R250
*HSPD1*	2	P10809	R121; R221
*LGALS1*	2	P09382	R19; R21
*ANXA11*	2	P50995	R230; R412
*CENPF*	2	P49454	R264; R3063
*B2M*	2	P61769	R32; R101
*GPI*	2	P06744	R81; R461
*TRIM21*	2	P19474	R84; R177
*ANXA5*	1	P08758	R6
*SNRPN*	1	P63162	R49
*CAPNS1*	1	P04632	R145
*DLAT*	1	P10515	R155
*CALR*	1	P27797	R177
*PHB1*	1	P35232	R195
*RACK1*	1	D6R9Z1	R231
*PHB2*	1	Q99623	R270
*NIN*	1	Q8N4C6	R1527

To explore the dynamic turnover rate of citrullination at individual sites, we next used a GSK484 inhibitor to investigate the kinetics of citrullination at the proteome-wide scale. By evaluating the reduction of citrullination globally and cumulatively at the site level, we found that a GSK484 concentration value of 1–3 µM reduced citrullination by 50% (Fig. 3e) (Supplementary Note 2).

Further investigation revealed that the half-maximal inhibitory concentration (IC_50_) value for GSK484 on the known regulatory autocitrullination of PADI4 (R218) is less than 1 µM (Fig. 3f). Looking at additional autocitrullination of PADI4, we found aberrant citrullination events on the less-preferred autocitrullination sites R372 and R374, as previously reported^46^. However, owing to the overall lower abundance of these modification sites, a proper IC_50_ value could not be established across the investigated cell conditions. Still, our data shed light on the regulatory effect that PADI4 inhibitors could have on citrullination of proteins, including β-actin (Fig. 3g), enolase (Extended Data Fig. 3g) and myelin basic protein (MBP) (Extended Data Fig. 3h)^4,47,48^, that are commonly citrullinated in autoimmune diseases.

### Histone citrullination

PADI4 is a well-established regulator of gene expression by modifying histone proteins through citrullination^49,50^. Additionally, histone hypercitrullination is known to have a critical role in chromatin decondensation in neutrophils^33^. Hence, our data could offer global insights into PADI4-mediated histone citrullination events.

First, we observed that 12–14% of the total cellular citrullination signal in NLCs localizes to histone proteins (Extended Data Fig. 3i), even when the global citrullination signal is decreased upon GSK484 treatment. By contrast, histones occupy only ~3% of the total protein signal in cells (Extended Data Fig. 3i), demonstrating that core histones are major citrullination targets in human cells.

Overall, we achieved wide sequence coverage of the histone backbones, including the amino-terminal regions (Extended Data Fig. 3j). We identified citrullination sites across all four core histones, with the highest global citrullination signal observed in the case of H3 (Fig. 3h) (Supplementary Table 4). Generally, the histone citrullination signal increased upon calcium stimulation, and conversely was reduced upon PADI4 inhibition, albeit to differing degrees and with different site specificities. The degree of regulation was pronounced for a range of citrullination sites (Fig. 3i), with the most abundant sites detected on H3, H1 and H2A. For example, citrullination of H3R26, H1R53, H2AR42 and H3R17 sites occupied 50%, 13%, 10% and 6%, respectively, of the total histone signal, and GSK484 reduced citrullination at all these sites by >80% (Supplementary Table 3; see Supplementary Note 3 for comment on the modest reduction of citrullination of some markers, such as H3R8, following GSK484 treatment). In our analysis, we quantified nearly all currently known histone citrullination sites while expanding on the current repertoire, enabling us to obtain a comprehensive map of endogenous PADI4-catalyzed histone citrullination marks (Fig. 3i). Although anti-citrullinated H2B antibodies are observed in 90% of people with RA^51^, we found that H2B citrullination occupies <1% of total histone citrullination levels (Fig. 3h), suggesting that even low-abundance histone citrullination marks may constitute an antigenic target of the ACPA immune response. The IC_50_ values for H1, H2A and H3 were also estimated (Extended Data Fig. 3k–m).

Collectively, these results provide the first global map of the PADI4-regulated histone sites in mammalian cells, demonstrating that PADI4 targets all four core histones but exhibits varying degrees of regulation, site-specificity and inhibition of citrullination across individual sites and histones. Still, further investigations into the site-specific histone citrullination landscape are likely to enable a mechanistic understanding of PADI4-mediated citrullination and transcriptional regulation.

### PADI4 targets transcriptional regulators

**Fig. 4:**
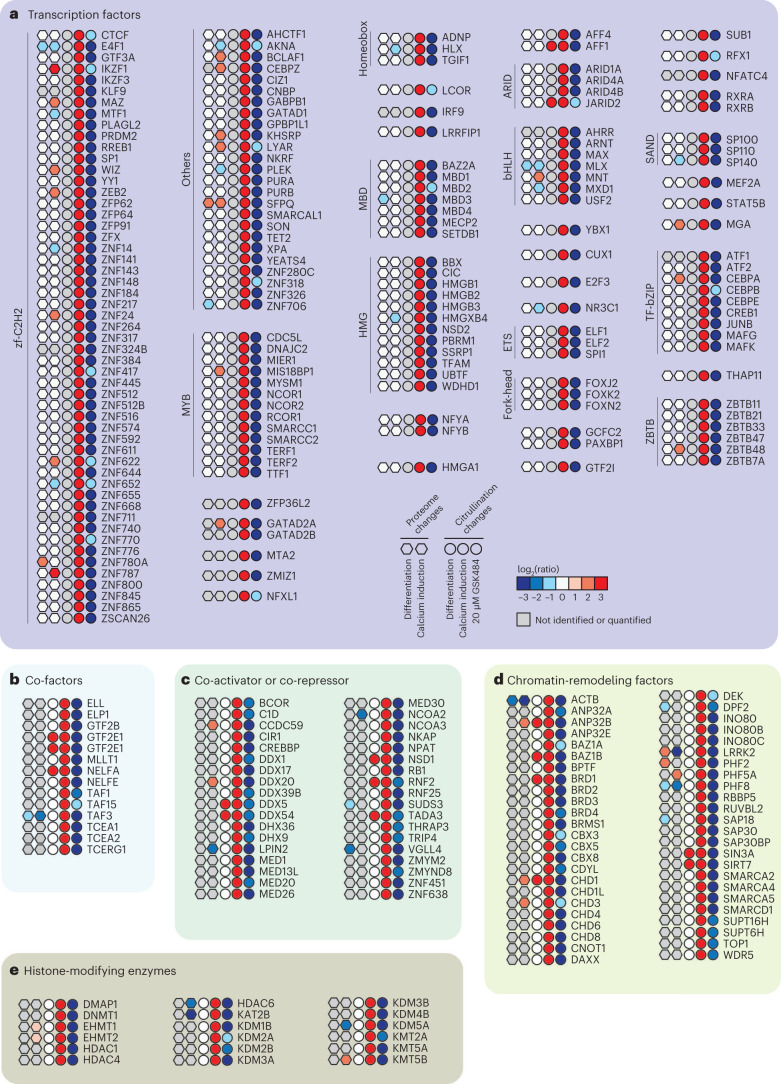
Regulation of transcription factors and other factors by PADI4 activity. **a**–**e**, Proteome changes, indicated by color in hexagons, in response to DMSO-induced differentiation and calcium induction, and citrullination changes, indicated by color in circles, in response to differentiation, calcium induction or 20 μM GSK484, for transcription factors (**a**), co-factors (**b**), co-activators and co-repressors (**c**), chromatin-remodeling factors (**d**) and histone-modifying enzymes (**e**).

**Extended Data Fig. 4:**
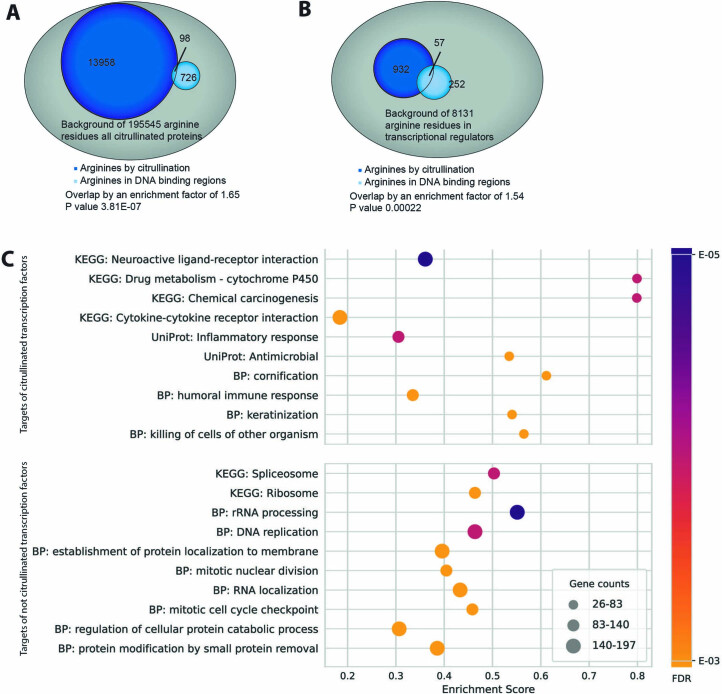
Further investigation of citrullination targeting. **a**) Venn diagram displaying significant overlap between arginines targeted for citrullination and arginines in DNA-binding regions across all arginines in target proteins according to two-tailed Fisher Exact testing with Benjamini-Hockberg correction, enrichment factor 1.65, p-value 3.81E-07. **b**) Venn diagram displaying significant overlap between arginines targeted for citrullination and arginines in DNA-binding regions across all arginines in transcriptional regulators citrullinated by PADI4 according to two-tailed Fisher Exact testing with Benjamini-Hockberg correction, enrichment factor 1.54, p-value 0.00022. **c**) Annotations enriched for the targets of citrullinated (top) and not citrullinated TFs (bottom) from the categories KEGG pathways, Gene Ontology Biological Process (BP), and UniProt keywords as determined by STRING gene-set enrichment analysis (see Supplementary Tables 6 and 7) for a list of all enriched annotations). Gene count refers to the number of genes annotated with the given annotation term, the false discovery rate (FDR) indicates the significance for each term, and enrichment score is the ratio between the term mean and the maximum deviation from the mean in the user input, multiplied by a factor of 10.

Although PADI4 is a known regulator of gene expression, and a regulatory function of citrullination of non-histone proteins has been described^52–54^, insight into the site-specific citrullination of transcriptional regulators remains limited. In our data set, we uncovered citrullination of >300 transcription factors, chromatin remodelers, histone-modifying enzymes and transcriptional co-activators (Fig. 4); 179 exhibited increased citrullination upon calcium stimulation and, conversely, decreased citrullination upon calcium stimulation and GSK484 treatment, indicating that citrullination of these proteins is mediated by PADI4. This corroborates the notion that PADI4 acts at the nexus of a broad range of transcription regulatory pathways and supports earlier findings from genome-wide analyses of PADI4 activity that linked the enzyme to active genes and supported its role as a regulator of gene expression^49,50,55^. We further found that PADI4 has a significant preference for targeting DNA-binding regions of the transcriptional regulators (Extended Data Fig. 4a,b and Supplementary Note 4). The downstream targets of the citrullinated transcription factors were investigated (Extended Data Fig. 4c and Supplementary Note 5), providing an example of how our data set can be used to generate hypotheses regarding the potential biological implications of citrullination.

### Properties of the PADI4-dependent citrullinome

**Fig. 5:**
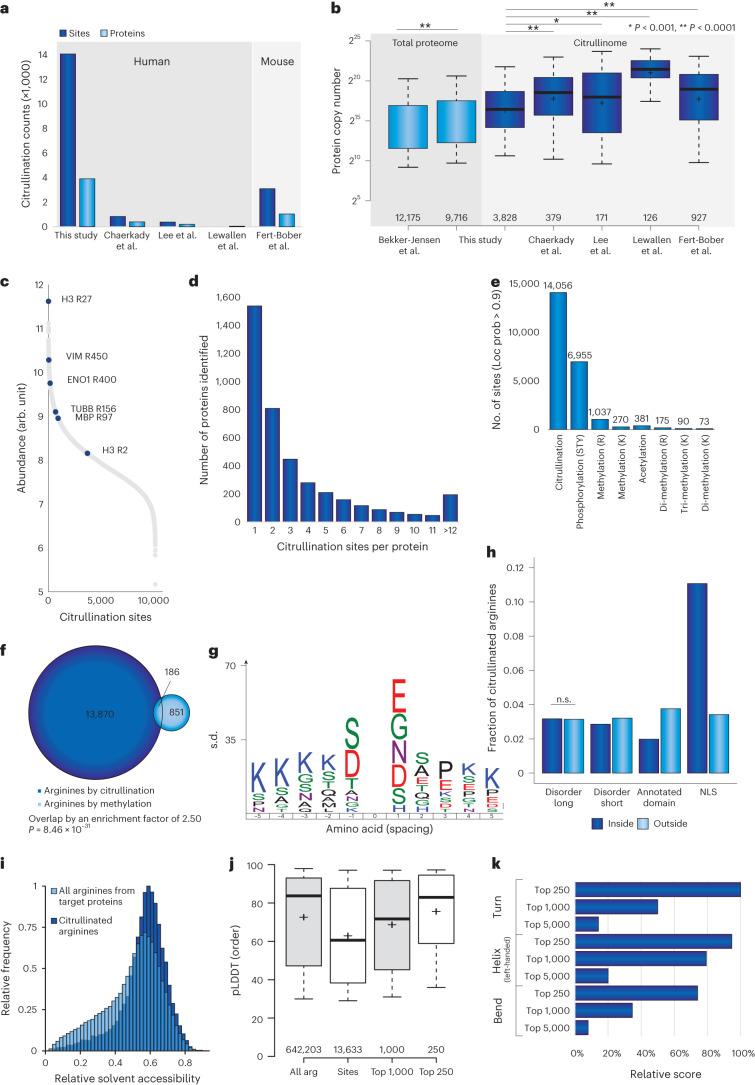
Properties of the citrullinome. **a**, Number of identified citrullination sites and citrullinated proteins in this study and others^27^^,30^^,120,121^. **b**, Protein copy number of proteomes (light blue) and citrullination screens (dark blue) in our study and others^27,30^^,120,121^ relative to deep proteome published by Bekker-Jensen et al.^28^. Whiskers, 95th and 5th percentiles; box limits, third and first quartiles; center bar, median; ‘+’ symbol, average. *n* is listed below the bars. Significance was determined by two-tailed Student’s *t* testing, with no correction for multiple comparisons. *P < 0.001, **P < 0.0001. *P* values from left to right, 3.91 × 10^–40^, 1.97 × 10^–101^, 2.03 × 10^–27^, 0.00057 and 3.53 × 10^–54^. **c**, Distribution of abundance of all sites detected. The most abundant sites on known citrullinated self-antigens are indicated across the distribution. **d**, Number of citrullination sites per protein. **e**, Number of common PTMs detected in our data set. **f**, Pie chart showing the distribution of arginines exclusively targeted by methylation and arginines co-targeted by methylation and citrullination. **g**, IceLogo representation of the enriched amino acid sequence context surrounding identified citrullination sites in the DMSO+Cal30' condition, as detected by Exploris, compared with sequence context of all arginines detected in the proteome from the study. Amino acid height corresponds to s.d. change. All displayed amino acids represent significant changes, as determined by two-tailed Student’s t-testing, *n* = 6,245 serine ADP-ribosylation sites, *P* < 0.05. **h**, Analysis of citrullination sites within various protein features reveals a significant enrichment in the localization of citrullination sites with the nuclear localization signal of proteins. **i**, Histogram showing relative solvent accessibility of citrullinated arginines and all arginines from the same target proteins. Two-tailed Spearman nonparametric correlation analysis shows a significant difference (7.81 × 10^–25^). **j**, Boxplot displaying predicted local distance difference test (pLDDT) order, derived from AlphaFold, of all global arginine residues (All arg), citrullination sites (Sites), the top 1,000 most abundant citrullination sites and the top 250 most abundant citrullination sites. Whiskers, 95th and 5th percentiles; box limits, third and first quartiles; center bar, median; ‘+’ symbol, average. *n* is listed below the bars. **k**, Relative score from the Fisher exact test of different secondary structures from AlphaFold, Turn, Helix left-handed and Bends, showing increased enrichment in response to citrullination abundance. Source data

**Extended Data Fig. 5:**
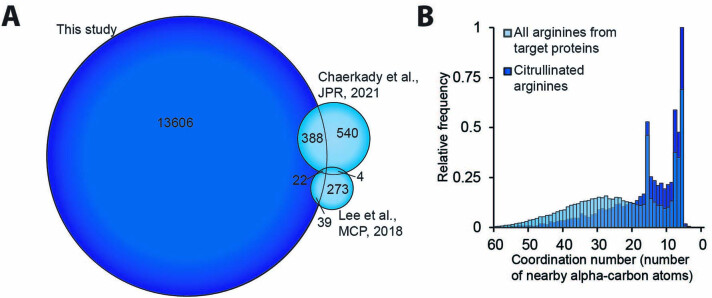
Comparison to previous studies and coordination number of arginines. **a**) Scaled venn diagram displaying overall of sites detected in our study, by Chaerkady et al., 2021 and Lee et al., 2018. **b**) Histogram showing coordination number (number of nearby alpha-carbon atoms) of citrullinated arginines and all arginines from the same target proteins. Two-tailed Spearman nonparametric correlation analysis show significant difference (7.53e-032).

Collectively, our large-scale analysis resulted in the high-confidence identification of 14,056 citrullination sites on 4,008 proteins (Fig. 5a), expanding the current knowledge regarding citrullination sites by 16-fold (Fig. 5a). Overall, the citrullination sites identified in this study largely overlap with those found in previous studies (Extended Data Fig. 5a).

To assess the depth of sequencing, we compared our study with a comprehensive study of the human proteome^28^. To this end, we plotted the measured intensity-based absolute quantification (iBAQ) values of the deep human HeLa proteome and aligned these with the corresponding values for the identified citrullinated proteins and the NLC proteome (Fig. 5b). Although we achieved comparable depth of sequencing for our NLC proteome analysis, the citrullinome analysis remained less sensitive, which is not surprising considering the overall stoichiometry of citrullination sites (Fig. 2h). Still, when assessing the depth of sequencing afforded by our data, the detected citrullination sites span seven orders of magnitude in terms of cellular abundance (Fig. 5c), in line with the pattern of other widespread modifications and supporting the excellent sensitivity achieved by our proteomics approach.

On average, citrullinated proteins harbored 3.4 citrullination sites. Only 1 citrullination site was detected in 40% of proteins (Fig. 5d), 431 proteins harbored >10 citrullination sites and 26 proteins were citrullinated on >20 arginine residues (Supplementary Table 4). The distribution of citrullination across targeted substrates is relatively similar to those of other widespread protein modifications, such as phosphorylation^56^, arginine methylation^57^ and SUMOylation^58^.

Citrullination and arginine methylation are described as functionally interacting on histones in the context of epigenetics and regulation of splicing factors^59,60^. However, little is known as to whether the two modifications occupy the same arginine residues within proteins. To assess this, we first analyzed our data for peptides modified with various classes of PTMs, such as phosphorylation, acetylation and various lysine and arginine methylation isoforms (Fig. 5e). In total, we were able to find 6,955 phosphorylation, 381 acetylation and 1,645 methylation forms, of which the majority (1,037) were identified as arginine mono-methylation sites. Notably, 186 citrullination sites overlapped site-specifically with identified arginine mono-methylation sites, a significantly higher degree of overlap than what is expected by chance (*P* < 3 × 10^–107^) (Fig. 5f). Thus, we show that citrullination and arginine mono-methylation show a statistically significant overlap on the same arginine residues on a proteome-wide scale.

Next, we identified a mixed sequence motif for targeting by PADI4 (Fig. 5g), including aspartic acid and serine at position –1 and aspartic acid and glycine at +1, as previously reported^30^, alongside other amino acids at +1 and –1. The motif is not as strong as detected for other PTMs; however, this may be due to differences in detection strategies.

**Fig. 6:**
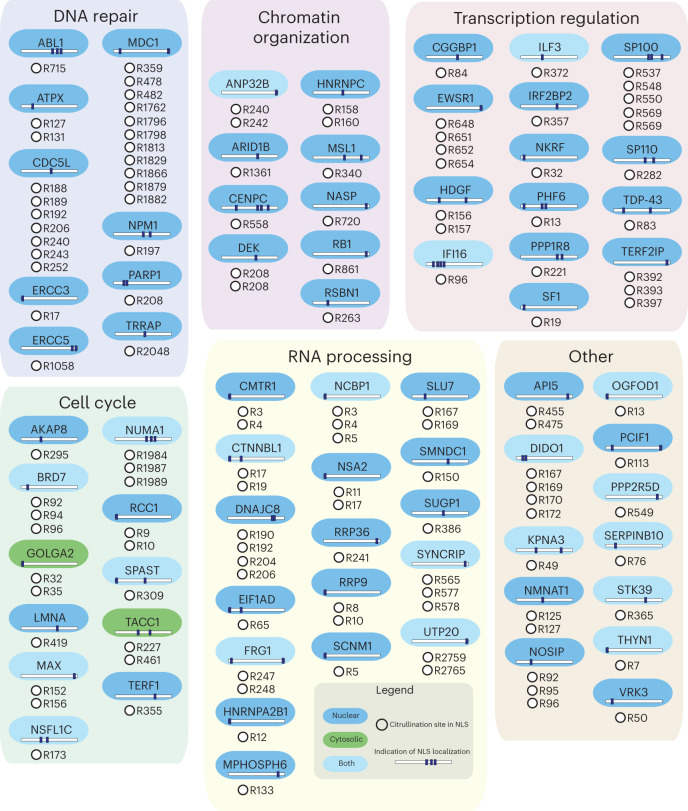
Targeting of nuclear localization signals. Citrullination site number overview. Proteins have been divided into functional families: DNA repair (top left), chromatin organization (top middle), transcription regulation (top right), cell cycle (bottom left), RNA processing (bottom middle) and other (bottom right).

Considering the large number of identified citrullination sites, we aimed to test whether citrullination sites localize to specific domains or structural regions of the modified proteins. We found that there is an enrichment for citrullination outside short disordered regions and annotated domains (Fig. 5h), which matches a previous report of citrullination preferentially targeting intrinsically disordered protein regions^3^. Additionally, we identified a strong enrichment for citrullination inside the nuclear localization signal (NLS) of proteins (Fig. 5h), along with a predominant targeting of factors related to nuclear processes, including DNA damage, chromatin organization, transcriptional regulation and the cell cycle (Fig. 6). This supports the notion that citrullination has an unappreciated role in regulating nuclear shuttling and protein localization. Consistent with this notion, we found that citrullination sites of PARP1 (R208), NPM1 (R197) and TDP-43 (R83) are targeted to arginine residues within their respective NLS domains, which are known to regulate the cytoplasmic translocation of these proteins^61–63^. We further investigated the relative solvent accessibility (RSA) of the citrullination events, to explore whether citrullination primarily occurs on buried or exposed residues. We found that citrullination occurs on exposed arginine residues, evidenced by a significantly higher RSA among the arginine residues targeted for citrullination than for all other arginine residues in the targeted proteins^64^ (Fig. 5i; *P* = 7.81 × 10^–25^). This is supported by a significantly lower number of nearby alpha-carbon atoms for the citrullinated arginine residues than for all arginine residues in the targeted proteins (Extended Data Fig. 5b; *P* = 7.53 × 10^–32^).

Finally, we aligned the citrullination sites with the AlphaFold database and, on the basis of the local distance difference test, observed that sites tended to localize within lower-order regions and primarily disordered regions, as compared with arginine residues, across all citrullinated proteins. However, upon considering more abundant citrullination sites, we observed an increase in structural order as the abundance increased (Fig. 5j). Furthermore, we investigated which secondary structures are preferentially targeted by PADI4 and found enrichment of bend, left-handed helices and turns among the most abundant citrullination events (Fig. 5k), whereas citrullination globally targets disordered regions (Fig. 5j). Collectively, citrullination preferentially targets exposed arginine residues and disordered regions on a global scale. However, the most abundant citrullination sites occur in regions of increased structural order.

### Validation of citrullination sites by peptide microarray

To highlight the value of our proteomics screen, we synthesized all identified citrullinated sites onto peptide microarray chips and assessed whether they were recognized by ACPAs from people with RA.

**Fig. 7:**
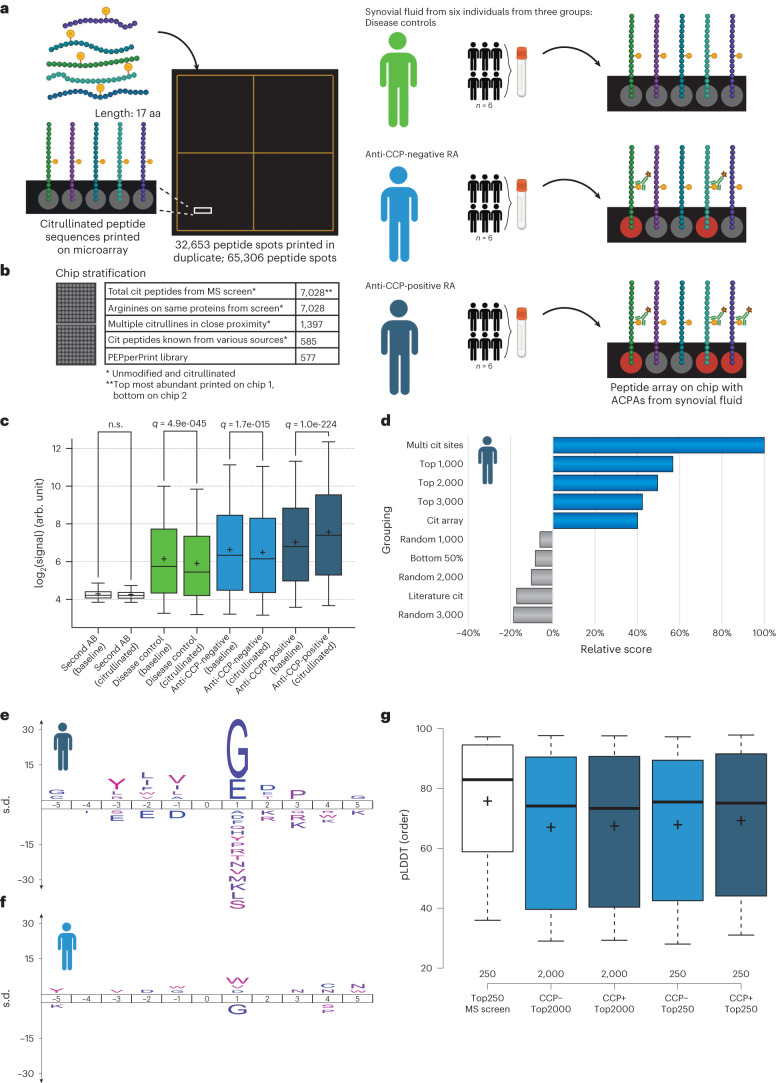
Validation of mapped citrullination sites by peptide microarray. **a**, Representation of workflow. Citrullination sites, detected by the MS screen, were printed on a microarray chip with the citrullination site centered in a 17-amino-acid peptide. Identical microarray chips with the citrullinated peptides and controls were then exposed to synovial fluids from three groups: (1) people with ankylosing spondylitis, (2) people with RA who were negative for anti-CCP antibodies and (3) people with RA who were positive for anti-CCP antibodies. Fluids were combined from six individuals in each group. The binding of the ACPAs to the citrullinated and control peptides was analyzed. **b**, Overview of peptides on the microarray. Owing to the limited capacity, the two first categories were split onto two chips. The other controls were included on both chips. **c**, Within-chip analysis of antibody binding in response to unmodified (baseline) peptide or citrullinated peptide. Second AB, secondary antibody binding. Whiskers, 95th and 5th percentiles; box limits, third and first quartiles; center bar, median; ‘+’ symbol, average. *n* = 65,306 peptide spots. **d**, Relative score from the Fisher exact testing of the antibody-binding response to multiple citrullinated sites (Multi cit sites); the top 1,000, 2,000 and 3,000 most abundant sites from the MS screen; PEPperCHIP cyclic citrullinated peptides (Cit array); 1,000 random sites; the bottom 50% responsive sites; 2,000 random sites; sites from the literature (Literature cit); and 3,000 random sites. **e**, Sequence motif of 1,000 singly modified citrullination sites mapped in the MS screen that overlap with best reactive sites from the anti-CCP-positive RA group; background is all other sites from the MS screen. **f**, Sequence motif of 1,000 singly modified citrullination sites mapped in the MS screen overlapping with reactive sites from the anti-CCP-negative RA group, compared to all other sites. **g**, Boxplot displaying pLDDT order, derived from AlphaFold, of the top 250 most abundant citrullination sites from the MS screen as a reference, the top 2,000 most reactive sites from the anti-CCP-negative RA group (CCP–), the top 2,000 most reactive sites from the anti-CCP-positive RA group (CCP+), the top 250 most reactive sites from the anti-CCP-negative RA group and the top 250 most reactive sites from the anti-CCP-positive RA group. Whiskers, 95th and 5th percentiles; box limits, third and first quartiles; center bar, median; ‘+’ symbol, average. *n* is listed below bars.

To this end, we obtained synovial fluid samples from people with RA who were either negative or positive for anti-CCP antibodies, a proxy for ACPAs, and investigated their capacity to interact with the citrullinated sites we identified in our MS screen (Fig. 7a). Synovial fluid from individuals with ankylosing spondylitis was used as a non-RA control. In addition to the identified citrullination sites, our microarray chip featured peptide sequences encompassing the multi-citrullinated peptides we identified, their corresponding non-modified peptide variants, random arginine residues derived from the same proteins as the identified sites, peptides known to be citrullinated in the literature^65–67^ and a commercially available PEPperPRINT CCP array (linearized) (Fig. 7b). In total, we synthesized duplicates of 32,653 peptide sequences on the microarray chip, resulting in a total of 65,306 sequences. The intensity derived from antibody binding to these citrullinated peptides was compared with that of the binding to their unmodified (baseline) variants on the same chip.

**Extended Data Fig. 6:**
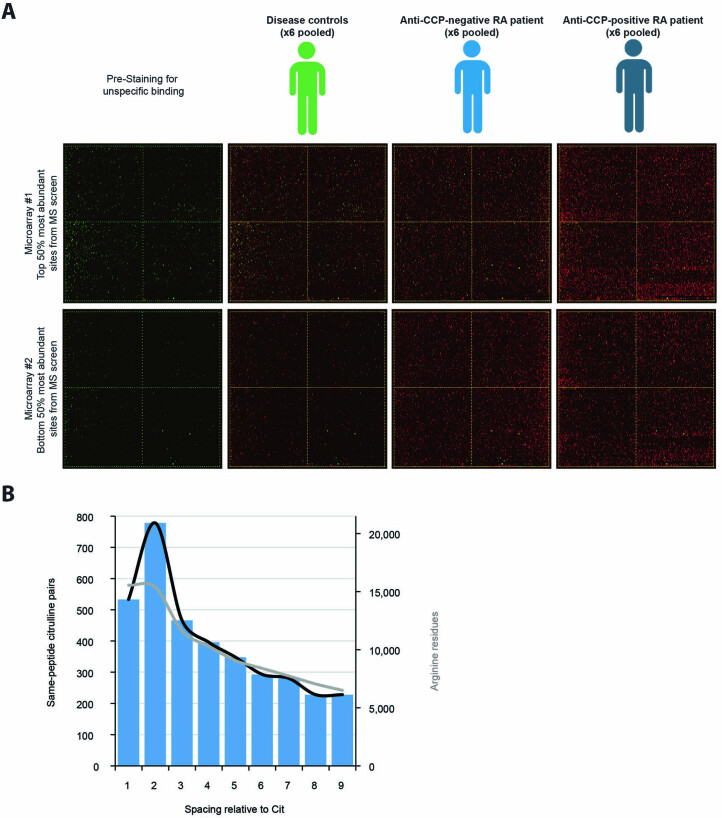
Raw images of peptide microarrays and peptide spacing. **a**) Raw peptide microarray images of microarrays, column 1 depicts microarray #1 and #2 prestained with secondary antibody Goat anti-human IgG (Fc), column 2 microarrays incubated synovial fluid from disease controls, column 3 microarrays incubated with synovial fluid from CCP- RA patients and column 4 microarrays incubated with synovial fluid from CCP+ RA patients. **b**) Distance of same-peptide citrulline pair spacing (black), arginine spacing indicated (grey).

Using this array, we detected autoantibodies in all three groups. As expected, only in the anti-CCP-positive RA group did the level of binding to the citrullinated form of the antigens exceed that of the binding to the baseline peptides (Fig. 7c). We noted that there were low background signals from the secondary antibody control, suggesting that the binding of autoantibodies was antigen-specific in all cases. Additionally, in the raw images used for quantification (Extended Data Fig. 6a), we noted that while the control samples do show some binding of antibodies, the binding is stronger in the wells containing synovial fluid from people with RA, in particular in those containing synovial fluid from those who are the anti-CCP-positive.

Upon closer examination, we noticed that the controls and the anti-CCP-negative RA group overall demonstrated increased antibody reactivity to the unmodified (baseline) peptides, whereas the anti-CCP-positive RA group exhibited stronger reactivity, particularly towards citrullinated peptides (Fig. 7c). This observation supports the notion that the ACPA binding in the anti-CCP-positive group is more specific towards the citrullinated sites identified in our MS screen.

Next, we investigated the potential impact of cellular abundance of citrullination sites, as detected in our MS screen, on overall ACPA binding. To achieve this, we correlated the peptide signal intensities obtained from our proteomics data with the ACPA binding levels observed in the array data. This analysis revealed a notably heightened response directed at citrullination sites with substantial cellular abundance (Fig. 7d). Furthermore, we observed a pronounced ACPA binding towards multi-citrullinated peptides, indicating that patches of citrullination in close proximity on proteins might elicit the strongest binding. Given that the existence of multiple citrulline sites within a single peptide could facilitate several simultaneous binding events from different ACPAs, we investigated the spacing between the sites to see if such a model would be permissive. Interestingly, the preferred spacing between citrullines on the peptide is short; just one amino acid separates the citrullinated residues for the most reactive peptides (Extended Data Fig. 6b), which makes simultaneous binding unlikely. This suggests that at least some ACPAs could have the capacity to recognize multi-citrullinated epitopes.

We then investigated the sequence motif for the strongest interactors in the two RA groups and found a strong preferential binding of ACPAs from the anti-CCP-positive RA group towards a Cit-Gly motif (Fig. 7e), which correlates with previously reported sequence motifs for ACPA recognition^65,66,68,69^. In addition to the Cit-Gly motif, we also found that positions –3 to –1 upstream of the Cit residue were enriched for bulky aliphatic amino acids, which create a hydrophobic patch that has not previously been described. In combination, these features underpin a recognition pattern that drives the specific ACPA binding for this anti-CCP-positive subgroup. Conversely, we observed an under-representation of glycine at the +1 position, and no preference for hydrophobic amino acids in the anti-CCP-negative RA group (Fig. 7f). This observation raises the possibility that a distinct antibody-specificity profile might underlie the lack of response to conventional anti-CCP tests in this group. Following the sequence motif analysis, we explored whether the most reactive sites in the anti-CCP-negative and anti-CCP-positive RA groups reside within regions of defined secondary structure, as was the case for the increasingly abundant citrullination sites during the MS screen (Fig. 5i). This was not the case for the top 2,000 and top 250 reactive peptides in the two groups, as the most reactive peptides target lower-order regions (Fig. 7g).

In conclusion, these results underscore the biological importance and potential clinical relevance of the identified citrullination sites, and their value as a resource to the field. We find that antibodies from people with RA that are classified as either anti-CCP-positive or anti-CCP-negative show a distinct preference for different citrullination motifs, and that multiple citrullination events that form a patch capture most ACPAs. We are also able to demonstrate that the ACPA binding for the anti-CCP-positive RA group is specific for the identified sites over baseline peptides.

## Discussion

We present a high-confidence atlas focusing on the PADI4-regulated citrullinome, characterizing more than 14,000 sites using high-accuracy mass spectrometry in the HL60 model system. Our findings expand current knowledge by 16-fold, offering insights into the biological implications of citrullination. Pinpointing citrullination sites across the proteome enhances the understanding of functional consequences, aiding the development of reagents for biochemical and cell-biology studies. Using the PADI4 inhibitor GSK488, we demonstrate widespread regulation of citrullination by PADI4, suggesting that the scope of PADI4 targeting is broader than anticipated. Our observations propose a paradigm of PADI4-mediated citrullination as a high-density ‘citrullination spray’ across various substrates, akin to other modifications such as SUMOylation and ADP-ribosylation^70^.

Considering the widespread interest in targeting PADI enzymes to treat a variety of human pathologies^71,72^, evaluation of the citrullinated proteome under treatment regimens with PADI-specific drugs, as demonstrated in this study, provides insightful information and reveals useful endogenous biomarkers to interrogate pathway function. For example, increasing evidence suggests that citrullinated versions of endogenous proteins constitute autoantigens in a variety of autoimmune disorders, and that corresponding ACPAs could serve as diagnostic and prognostic markers^73,74^. In support of this, our data detail the specific citrullination sites related to 44 autoantigens specific to RA.

Our data also provide insights into the specificity of PADI4 as a peptidylarginine deiminase, and we find that PADI4 citrullinates substrate proteins independently of linear sequence. We further find that citrullination is primarily directed to disordered regions, which supports a role for the process in modulating protein binding, considering that disordered protein regions hold central roles in protein interaction networks. Similarly, we find that citrullination localizes outside annotated domains, with the exception of the strong enrichment of citrullination sites inside the nuclear localization signal of proteins. Intriguingly, citrullination preferentially targets exposed arginine residues in disordered regions globally, but the most abundant sites occur in regions of increased structural order. GO analysis of the genes regulated by citrullinated transcription factors shows enrichment for pathways involved in immune responses and terms related to the maintenance of the skin barrier, areas in which physiological citrullination is known to have a direct role^33,75–77^. These results not only offer indirect insights, but also highlight the rich information within our data, suggesting that exploration of the functional role of citrullination beyond individual sites could be fruitful. Although in-depth studies are needed to comprehend PADI4’s regulatory role in gene regulation, our research contributes to a systems-wide understanding of the connection between citrullination and gene transcription. Additionally, we reveal a notable overlap in arginine targeting by citrullination and methylation, supporting previous indications of a potential inhibitory interplay between these two post-translational modifications, both recognized for their roles in regulating transcriptional activity.

Histone modifications are widely linked to transcriptional regulation, and great efforts have been made to map the genomic regions targeted by various histone modification marks^78^. In regard to citrullination, however, the understanding of the enzymatic specificity and dynamics remains limited^79^. Within our MS-based quantitative atlas, we quantify histone citrullination sites exclusively catalyzed by PADI4, which could help to more accurately define genomic regions that are occupied by catalytically active PADI4, especially considering that epigenetic mapping of histone marks typically focuses on histones H3 and H4.

Our kinetic analyses of PADI4 inhibition provide the first proteome-wide survey of site-specific citrullination events directly mediated by PADI4 through identification of a large set of sites that are downregulated upon treatment with GSK484. This regulation is reminiscent of the inhibition of other enzymatic regulations within, for example, phosphorylation and acetylation signaling^80,81^. Our results demonstrate that PADI4-mediated citrullination signaling is regulated to a similar degree as other widespread modifications.

To demonstrate the utility of our citrulline atlas for biological insights, we investigated how synovial fluid antibodies from people with RA react to the identified sites, given the autoimmune response directed at citrullinated antigens in RA, especially in people with anti-CCP antibodies^21,82,83^. To this end, we synthesized all identified citrullination sites as linear peptides on a microarray. Upon testing whether the sites were recognized by autoantibodies in synovial fluid samples, we observed binding in all groups; the anti-CCP-positive group exhibited stronger binding, consistent with clinical severity^84–88^. The decreased antibody binding towards citrullinated peptides in the disease control and anti-CCP-negative groups (Fig. 7c) might be due to disruption of native binding epitopes lacking citrulline. Although our proteomics data did not reveal a defined sequence motif, our microarray data unveiled a recognition motif in anti-CCP-positive individuals, a hydrophobic patch followed by Cit-Gly that has not previously been described^66,68^. This suggests that PADI4’s catalytic activity is not directed toward a specific motif (Fig. 5g) but might instead act as a ‘citrullination spray’. Still, antibodies from anti-CCP-negative individuals with RA showed no preference towards Cit-Gly motifs, potentially explaining the challenges in their identification using contemporary anti-CCP tests biased toward detection of Cit-Gly motifs^89^.

Hence, we propose that the new motif identified in this study, [3Φ-Cit-Gly], where 3Φ denotes three bulky hydrophobic residues, may enhance RA diagnosis specificity. Additionally, the over-representation of multi-citrullinated peptides for binding suggests that the increased density of citrullination in a concentrated patch may improve the performance of the next-generation ACPA test for enhanced RA diagnosis.

In summary, our citrulline site atlas enhances our understanding of RA and could help improve RA diagnostic techniques. Citrulline is integral to several autoimmune disorders, including multiple sclerosis, lupus, psoriasis and inflammatory bowel disease^21^.

Unlike RA, in which ACPA reactivity is central, specific autoantibodies for these diseases are yet to be discovered. Therefore, our resource data could improve early diagnosis for individuals with autoimmune diseases by facilitating the discovery of new biomarkers.

In conclusion, this in-depth analysis provides a powerful resource for identifying and quantifying citrullinated residues induced by PADI4. It establishes a framework for decoding PADI4 functions in various biological processes and enhances our understanding of PADI4-specific inhibitors. This comprehensive resource allows for easy future investigation of individual site utilization.

## Methods

Our research complies with all relevant ethical regulations. The samples used in this study were obtained following informed consent from individuals recruited at the Center for Rheumatology and Spine Diseases, Copenhagen University Hospital Glostrup, after approval by the local ethical committee (approval ID H-16042831). The local ethics committee was: De Videnskabsetiske Komiteer for Region Hovedstaden, Regionsgården Kongens Vænge 2, 3400 Hillerød, Denmark.

### Cell culture

HL60 cells were grown in RPMI medium (cat. no. 21875091, Gibco) supplemented with 10% fetal bovine serum (FBS) and penicillin–streptomycin (100 U ml^–1^) (Gibco) at 37 °C and 5% CO_2_. The HL60 cell line was a gift from M. Christophorou. A proportion of the cells were differentiated over the course of 4 d to neutrophil-like cells by the addition of 1.25% (vol/vol) DMSO to the medium. The experiments were performed in biological (cell culture) triplicate. Cells were washed in 37 °C PBS and transferred to 37 °C Locke’s solution (10 mM Hepes pH 7.5, 150 mM NaCl, 5 mM KCl and 2 mM CaCl_2_, 0.1% glucose) at a concentration of 2 × 10^6^ cells ml^–1^. Citrullination of proteins was induced via activation of PADI4 by the addition of calcium ionophore Af23187 (cat. C7522, Merck) to a final concentration of 4 µM, for either 15 or 30 min, at 37 °C. Control samples, of both undifferentiated HL60 cells and differentiated neutrophil-like cells, were collected from Locke’s solution before calcium treatment for further sample preparation.

### PADI4 inhibition by GSK484

Quadruplicate cultures of NLCs were incubated in Locke’s solution, as described above, with a range of GSK484 (cat. no. SML1658, Merck) concentrations at 37 °C for 30 min before calcium activation, using 4 µM calcium ionophore A23187 (cat. no. C7522, Merck). The GSK484 concentrations were 1 µM, 5 µM and 20 µM, in addition to a DMSO control.

### Cell lysis and protein digestion

The cell pellets were lysed in lysis buffer (6 M guanidine-HCl, 50 mM TRIS, pH 8.5) and further processed using standard sample-preparation methods. The proteins were digested by two rounds of Lys-C digestion (see Supplementary Note 6 for details).

### Purification of peptides

Peptides were purified using reversed-phase C18 cartridges (SepPak Classic, 350 mg, Waters). Cartridges were activated with 5 ml acetonitrile (ACN) and equilibrated three times with 5 ml of 0.1% TFA, after which samples were loaded. Sample loading was accelerated using a vacuum manifold, maintaining two-thirds atmospheric pressure. Following loading, cartridges were washed three times with 5 ml of 0.1% TFA, after which peptides were eluted using 4 ml of 30% ACN in 0.1% TFA. The eluted peptides were frozen overnight at −80 °C in 15-ml tubes with small holes punctured into the caps, after which the frozen peptides were lyophilized for 96 h. Lyophilized peptides were dissolved in 25 mM ammonium bicarbonate pH 8.5, and the peptide concentration was estimated through absorbance at 280 nm, using a NanoDrop instrument.

### Offline high-pH reversed-phase HPLC fractionation

For each experimental replicate, 0.6 mg peptide was fractionated into 46 fractions using an XBridge BEH130 C18 3.5 µm 4.6 mm × 250 mm column (Waters) on an Ultimate 3000 HPLC system (Dionex), operating at a flow rate of 1 ml min^–1^. The flow was composed of three buffers: buffer A (Milli-Q water), buffer B (100% ACN) and buffer C (25 mM ammonium hydroxide). Prior to loading, samples were basified to pH > 10 by the addition of ammonium hydroxide (gradient details are available in Supplementary Note 7). Collected fractions were transferred to Eppendorf Protein LoBind tubes with small holes punctured in the caps, and frozen at −80 °C overnight. The frozen samples were lyophilized for 96 h and afterwards were dissolved in 1% formic acid (FA).

### Mass spectrometry analysis

Samples were measured using a Q-Exactive HF-X mass spectrometer or an Exploris 480 mass spectrometer (Thermo Fisher Scientific). Peptides were separated by online reversed-phase liquid chromatography using an EASY-nLC 1200 system (Thermo Fisher Scientific), using a 15-cm-long analytical column with an internal diameter of 75 µm, packed in-house using ReproSil-Pur 120 C18-AQ 1.9 µm beads (Dr. Maisch). The analytical column was heated to 40 °C using a column oven, and peptides were eluted from the column using a gradient of buffer A (0.1% FA) and buffer B (80% ACN in 0.1% FA). The gradient ranged from 4% to 38% buffer B over 30 min, followed by an increase to 90% buffer B over 4 min to ensure elution of all peptides, followed by a washing block of 6 min.

For the pilot experiment, performed on the Q-Exactive HF-X instrument, electrospray ionization was achieved using a Nanospray Flex Ion Source (Thermo Fisher Scientific). The spray voltage was set to 2 kV, the capillary temperature to 275 °C and the radio frequency level to 40%. Full scans were performed at a resolution of ×60,000, with a scan range of 300 to 1,750 *m/z*, a maximum injection time of 60 ms and an automatic gain control (AGC) target of 3,000,000 charges. Precursors were isolated at a width of 1.3 *m/z*, with an AGC target of 200,000 charges. Repeated sequencing of selected precursors was excluded by dynamic exclusion of 60 s. Precursor fragmentation was achieved using higher energy collision dissociation (HCD). MS/MS data were measured using the Orbitrap with a maximum injection time of 90 ms and a resolution of 4×5,000. The Top9 data-dependent MS/MS method was used to acquire MS data.

For the optimized GSK experiment, performed on the Exploris 480 instrument, electrospray ionization was achieved using a NG Ion Source (Thermo Fisher Scientific). The spray voltage was set to 2 kV, the capillary temperature to 275 °C and the radio frequency level to 40%. Full scans were performed at a resolution of ×120,000, with a scan range of 300 to 1,750 *m/z*, the maximum injection time set to ‘Auto’ and the normalized AGC target set to ‘200’ (2,000,000 charges). Precursors were isolated at a width of 1.3 *m/z*, with a normalized AGC target of ‘200’ (200,000 charges). Repeated sequencing of selected precursors was excluded by dynamic exclusion of 60 s. Precursor fragmentation was achieved using HCD. MS/MS data were measured using the Orbitrap with a maximum injection time set to ‘Auto’ and a resolution of ×30,000.

### Western blotting

Cell pellets were lysed in SDS Lysis Buffer (2% SDS, 50 mM Tris-HCl pH 8.5, 150 mM NaCl) and homogenized by heating to 99 °C and shaking at 1,400 r.p.m. for 30 min. Protein concentrations across lysates were equalized using the Pierce BCA Protein Assay Kit (cat. no. 23225, Pierce), according to the manufacturer’s instructions. An immunoblot was performed using standard approaches with an Invitrogen chamber and blot module. Proteins were transferred to a PVDF membrane (Immobilon) for 90 min at 0.4 A. Membranes were blocked using 5% milk (Fluka Analytical) in PBS supplemented with Tween-20 (0.1%; PBST) or 5% bovine serum albumin (BSA) in PBST, following the antibody manufacturer’s recommendations. The following antibodies were used: rabbit polyclonal PADI4 (1:1,000, cat. no. P4749, Sigma Aldrich), rabbit monoclonal H3 (citrulline Arg2) antibody (1:1,000, cat. no. Ab176843, clone EPR17703, Abcam) and rabbit polyclonal GAPDH (1:1,000, Ab9485, Abcam). The Anti-Citrulline (Modified) Detection Kit (cat. no. 17-347B, Merck) with the anti-modified citrulline antibody (part number MABS487, clone C4) was used to measure global citrullination^24^, according to the manufacturer’s instructions.

### Mass spectrometry data analysis

The raw mass spectrometry data files were analyzed using MaxQuant software (version 1.5.3.30), a freely available software routinely used in this field. MaxQuant settings used for analysis are available in Supplementary Note 8. A HUMAN.fasta database was extracted from UniProt on 5 May 2020 to serve as a theoretical spectral library. The HUMAN.fasta database contained 96,821 protein entries.

### Mass spectrometry data filtering

In addition to automatic filtering and FDR control, as applied by MaxQuant, the data were manually filtered to ensure proper identification and localization of citrullination. Citrullination-site identification was allowed only if the localization probability was >0.90. For quantification of citrullination, further PSMs were accepted with a localization of >0.75, as this is the standard cut-off in proteomics; the 0.90 used in this study for qualification is stringent. MaxQuant intensity values (a quantitative metric corresponding to peak area-under-the-curve at the MS1 level) were manually transferred from the evidence file (by mapping on experiment and fixed evidence IDs) to the citrullination sites using only PSMs with a localization of >0.75.

LFQ intensities were normalized within each condition and missing values were imputed across replicates using Perseus software.

### Mass spectrometry statistical analysis

Statistical handling of the data and hierarchical clustering was primarily performed using the freely available Perseus software (version 1.6.14.0)^92^. Significantly enriched Gene Ontology terms were determined using the Functional Annotation Tool of the DAVID Bioinformatics database^93,94^. Venn diagrams were generated using the online DeepVenn program^95^. Boxplots were generated using the BoxPlotR web tool^96^. Kinase–substrate relationships were predicted using the online NetworKIN tool^97,98^. The sequence motif was generated using the iceLogo software (version 1.2)^99^, with background sequences extracted from non-citrullinated arginine residues in all citrullinated proteins.

### Analysis of transcription factor target genes and gene-set enrichment

For each citrullinated and non-citrullinated transcription factor (TF), all target genes were retrieved from TFEA.ChIP using the ReMap 2020 (ref. ^100^) and GeneHancer Double Elite^101^ data set. Data were available for 115 citrullinated TFs and 233 non-citrullinated TFs. For each of the 16,544 target genes with an Ensembl annotation, the fraction of citrullinated and non-citrullinated TFs that regulate it was determined (Supplementary Table 6). The final score was calculated as the logarithm of the ratio of citrullinated versus non-citrullinated fractions. This value is negative for a given target if there are more non-citrullinated TFs regulating the target than citrullinated ones, and vice versa. All targets were ordered by this value and the whole ranked list was used as input for the STRING v11 gene-set enrichment analysis^102^. The resulting enriched annotations with a FDR below 0.05 are provided in Supplementary Table 7. A given annotation describes the citrullinated TFs if it is enriched at the bottom of the input, and the non-citrullinated TFs if it is enriched at the top of the input.

### Enrichment analysis of regions targeted for citrullination

For the analysis of citrullination sites in disordered regions, disorder was predicted using IUPred2A^103^ for all identified sequences. The set of long disordered regions was obtained using IUPred2A’s long disorder option and a minimum region length of 31 consecutive residues with a prediction score of ≥0.5. Regions predicted using the short disorder option were retained if they contained 2 to 30 consecutive residues with a score of ≥0.5. Predictions from all identified sequences were included in the analysis of disordered regions.

Annotations of domains and NLSs were obtained in gff format from UniProtKB^104^, and their sequences were derived from the sequences identified in the MS run. All identified sequences with any kind of feature annotation were included in the domain and NLS analyses.

For each feature category (long and short disorder, domain, NLS), a Fisher’s exact test was performed, using the counts of unmodified arginine residues and citrullinated arginine residues inside and outside the features. Fold enrichment of citrullines inside features compared with citrullines outside features was calculated as:

$${FE}=\frac{{{Cit}}_{\mathrm{i}}}{{R}_{\mathrm{i}}}\frac{{R}_{\mathrm{o}}}{{{Cit}}_{\mathrm{o}}},$$

where *Cit*_i_ and *Cit*_o_ refer to the numbers of citrullines inside and outside features, respectively, and *R*_i_ and *R*_o_ refer to the total number of unmodified and modified arginine residues inside and outside features.

### Peptide microarray screen using clinical rheumatoid arthritis samples

Peptide microarrays were produced in collaboration with PEPperPRINT, according to their custom PEPperCHIP Discovery microarrays workflow. Briefly, linear peptides were synthesized step-wise onto a coated glass slide using a precision laser, allowing the incorporation of non-canonical amino acids, such as citrulline. The peptide arrays each contained 75,460 17-amino-acid peptides from the following groups: citrullinated sites and their unmodified counterparts, peptides with multiple citrullination sites on the same peptide and their unmodified counterparts, randomly selected arginine residues from the same protein groups and their citrullinated counterparts, known ACPA reactive peptide sequences and their unmodified counterparts^20,67,105–115^ and a commercially available PEPperPRINT CCP array (see Supplementary Tables 8–15). Owing to the number of controls included, the citrulline sites were split across two chips containing the top 50% sites and bottom 50% sites, respectively, as determined by intensities derived from our MS experiment.

The peptide microarrays were incubated with synovial fluids pooled from six individuals for each of the three following groups: anti-CCP-positive RA; anti-CCP-negative RA; and ankylosing spondylitis. All participants were recruited at Center for Rheumatology and Spine Diseases, Copenhagen University Hospital Glostrup, after informed consent was obtained and under approval by the local ethical committee (H-16042831). Of the 18 participants, 12 were females and 6 were males. Participant age at the point of sample collection ranged from 28 to 76 years old. Unspecific binding was tested by incubating the microarray alone with the secondary antibody used for primary antibody detection. Before incubation, arrays were blocked with Rockland blocking buffer MB-070, after which the synovial fluid was diluted in the buffer and was allowed to bind to the chip for 16 h at 4 °C under mild shaking at 140 r.p.m. After incubation, the arrays were washed three times with PBS supplemented with 0.05% Tween-20 after each incubation step.

To detect binding of clinical anti-citrulline protein antibodies, the intensity of the secondary antibody goat anti-human-IgG (Fc) DyLight680 (0.1 µg ml^–1^) was recorded using the LI-COR Odyssey Imaging System with the following parameters: scanning offset 0.67 mm, resolution 21 µm, scanning intensities of 8/8 (red 680 nm; green 800 nm).

To quantify the intensity of the resulting spots, 16-bit grayscale tiff files of the arrays were recorded and analyzed the images with PepSlide Analyzer (Sicasys software).

### Peptide microarray data analysis

To analyze the peptide microarray data, we used the raw data provided by PEPperPRINT (‘Raw Data’ tabs in Supplementary Tables 8–15). The ‘Raw Mean’ intensity was used for further processing, as this represents the most basic readout and prevents any ‘0’ reads, which would have to be imputed at a later stage. Within each array, the median was calculated for all reads corresponding to the same peptide sequences, which in most cases was for *n* = 2 (duplicate prints), but in some cases was *n* = 4, 6, 8 or 10 for peptides in multiple classes that were printed several times on the same chip. All values were log_2_-transformed, at which point the global median of the pairs of chips treated with the same synovial fluid were normalized to each other. Next, we calculated ratios of change for all pairs of citrullinated peptides versus their unmodified counterpart peptide. At this stage, we computed the median for the ratios of peptide pairs that existed on both chips for the same synovial fluid mixture. This process was applied to only the controls, because they were printed in technical duplicates on two chips (as well as technical duplicates within each chip, as outlined above). This resulted in four data points for each citrulline-baseline pair, stratified only by synovial fluid type: second antibody control, disease control, anti-CCP negative, and anti-CCP positive. Finally, we calculated the *z*-score for each peptide pair. Supplementary Note 9 contains details on the statistical analysis of microarray data.

IceLogo v1.3.8 was used to generate sequence logos. As a foreground, we used the top 1,000 citrullinated arginine residues, identified by MS, overlapping either with the most enriched arginine residues in the anti-CCP-positive group (as derived from the microarray data) or the most enriched arginine residues in the anti-CCP-negative group. As a background, the other ~13,000 MS-identified citrullinated arginine residues were used. The *P* value for iceLogo analysis was set at 5% (default), and the s.d. was used to visualize the magnitude of change.

### NetSurfP-3.0 structural predictions

The NetSurfP3.0 model^64^ was used to predict the RSA for all residues in proteins identified as citrullinated in this study. To this end, the sequences for all citrullinated proteins were submitted in small batches to the ‘NetSurfP - 3.0’ web service at the Technical University of Denmark. All batches were collated, and the predicted values for arginine residues were extracted. For statistical comparison of the data, all foreground arginine residues (that is those found to be citrullinated) were compared versus all background arginine residues (that is, all non-modified arginine residues from the same proteins). RSA values ranged between 0 and 1, and were grouped into bins of 0.02 for visualization. Significance was tested using two-tailed Spearman nonparametric correlation analysis.

### Prediction of accessibility of citrullination sites

The accessibility of the citrullination sites was estimated from the AlphaFold^116^, predicted human protein structure database. For each of the AlphaFold structures, the exposure of all arginine residues was found. The exposure was estimated as the coordination number (CN). The CN is the number of Cα atoms within a sphere around the Cα atom, and for this study, a radius of 13 Å was applied. The CN was calculated using the Python program hsexpo from the Bio.PDB library^117^ and collected in a data set. For statistical comparison of the data, all foreground arginine residues (that is, those found to be citrullinated) were compared with all background arginine residues (that is, all non-modified arginine residues from the same proteins). Significance was tested using two-tailed Spearman nonparametric correlation analysis.

### Structural analysis via AlphaFold

All published AlphaFold predictions (.cif files) for human proteins were downloaded^116^, the files were parsed to output a text file containing the protein Unipot ID, amino acid residue, position in sequence, pLDDT score, F number and assigned structure for every residue in the library, and pLDDT values (model confidence) and secondary structure elements were parsed out for all arginine residues. If the AlphaFold predictions consisted of overlapping segments (as is the case for large proteins), the median pLDDT was taken for any residues predicted across multiple segments. For secondary structure elements, if there was ambiguity, the most confidently predicted elements (going by pLDDT) were taken. Next, we defined two background groups, all arginines in human proteins, and only arginines in proteins mapped by MS to be citrullinated in this study. Out of all MS-identified sites, 13,633 sites could be aligned to the AlphaFold predictions, that is pLDDT and secondary structure elements could be derived for these 13,633 citrullinated arginine residues. The missing sites were either not predicted by AlphaFold, had a mismatching protein ID (because of UniProt database changes over time), were exclusive to an isoform (AlphaFold predicted only canonical entries) or did not match to an arginine (because of UniProt database changes over time). pLDDT values were visualized as boxplots for several selections of arginine residues, using BoxPlotR^96^ for visualization. For investigation of enrichment of specific secondary structure elements, the Fisher exact test was performed to compare groups of secondary structure elements to groups of citrullination sites, using either all arginines in human proteins or all arginines in citrullinated proteins as a background. Correction for multiple-hypotheses testing was performed using Benjamini–Hochberg adjustment of the *P* values.

### Reporting summary

Further information on research design is available in the Nature Portfolio Reporting Summary linked to this article.

## Online content

Any methods, additional references, Nature Portfolio reporting summaries, source data, extended data, supplementary information, acknowledgements, peer review information; details of author contributions and competing interests; and statements of data and code availability are available at 10.1038/s41594-024-01214-9.

### Supplementary information

Supplementary Information Supplementary Notes 1–9.

Reporting Summary

Supplementary Tables 1–15 **Supplementary Table 1** A list of all peptides identified in the pilot study (study 1) on the HF-Exactive system and the inhibitor study (study 2) performed on the Exploris system. **Supplementary Table 2** A list of all identified protein-coding genes. **Supplementary Table 3** A list of all unique identified citrullination sites in the pilot study (study 1) on the HF-Exactive system and the inhibitor study (study 2) performed on the Exploris system. **Supplementary Table 4** A list of all identified citrullination-targeted protein-coding genes. **Supplementary Table 5** Statistical information related to term enrichment analysis, as found in Figs. 1 and 2 and Extended Data Fig. 2, found in separate tabs in the Excel file. **Supplementary Table 6** Target genes ranked by their relative regulation by citrullinated or non-citrullinated TFs (input file for STRING gene-set enrichment uses column log_ratio_fraction and ensembl_gene_id). **Supplementary Table 7** Results from STRING gene-set enrichment for all categories of functional annotations. **Supplementary Table 8** Data from microarray 1 exposed to the synovial fluid pool from RA-negative disease controls, people with ankylosing spondylitis, including a peptide map (tab 1), intensity map (tab 2), top peptides and intensity plot (tab 3), mapping summary (tab 4) and raw data (tab 5). **Supplementary Table 9** Data from microarray 1 exposed to the synovial fluid pool from the anti-CCP-negative RA group, including a peptide map (tab 1), intensity map (tab 2), top peptides and intensity plot (tab 3), mapping summary (tab 4) and raw data (tab 5). **Supplementary Table 10** Data from microarray 1 exposed to the synovial fluid pool from the anti-CCP-positive RA group, including a peptide map (tab 1), intensity map (tab 2), top peptides and intensity plot (tab 3), mapping summary (tab 4) and raw data (tab 5). **Supplementary Table 11** Data from microarray 1 exposed to secondary goat anti-human-IgG (Fc) DyLight680 antibody for background testing, including a peptide map (tab 1), intensity map (tab 2), top peptides and intensity plot (tab 3), mapping summary (tab 4) and raw data (tab 5). **Supplementary Table 12** Data from microarray 2 exposed to synovial fluid pool from RA-negative disease controls, people with ankylosing spondylitis, including a peptide map (tab 1), intensity map (tab 2), top peptides and intensity plot (tab 3), mapping summary (tab 4) and raw data (tab 5). **Supplementary Table 13** Data from microarray 2 exposed to the synovial fluid pool from the anti-CCP-negative RA group, including a peptide map (tab 1), intensity map (tab 2), top peptides and intensity plot (tab 3), mapping summary (tab 4) and raw data (tab 5). **Supplementary Table 14** Data from microarray 2 exposed to synovial fluid pool from the anti-CCP-positive RA group, including a peptide map (tab 1), intensity map (tab 2), top peptides and intensity plot (tab 3), mapping summary (tab 4) and raw data (tab 5). **Supplementary Table 15** Data from microarray 2 exposed to secondary goat anti-human-IgG (Fc) DyLight680 antibody for background testing, including a peptide map (tab 1), intensity map (tab 2), top peptides and intensity plot (tab 3), mapping summary (tab 4) and raw data (tab 5).

### Source data

Source Data Fig. 1 Unprocessed western blots for Fig. 1b.

Source Data Fig. 1 Statistical source data for Fig. 1.

Source Data Fig. 2 Statistical source data for Fig. 2.

Source Data Fig. 3 Unprocessed western blots for Fig. 3b.

Source Data Fig. 3 Statistical source data for Fig. 3.

Source Data Fig. 5 Statistical source data for Fig. 5.

Source Data Extended Data Fig. 3 Statistical source data for Extended Data Fig. 3.

## Data Availability

The mass spectrometry proteomics data have been deposited to the ProteomeXchange Consortium through the PRIDE^118^ partner repository with the data set identifier PXD038702. Statistical source data for all main figures and Extended Data figures can be found in the provided source data files in addition to Extended Data tables. Databases used in no particular order: AlphaFold^116^, UniProtKB^104^, DAVID Bioinformatics^94^, AAgAtlas 1.0 (ref. ^45^), ReMap2020 (ref. ^100^) and GeneHancer Double Elite^101^. Source data are provided with this paper.
