# Supplementary material for: A quantitative and site-specific atlas of the citrullinome reveals widespread existence of citrullination and insights into PADI4 substrates
Source: Nat Struct Mol Biol. 2024 Feb 6;31(6):977–95. doi: 10.1038/s41594-024-01214-9 (PMC11189309; doi:10.1038/s41594-024-01214-9)
Supplement: Supplementary file 1 — Supplementary Notes 1–9. [file 41594_2024_1214_MOESM1_ESM.pdf]

nature structural & molecular biology

Article https://doi.org/10.1038/s41594-024-01214-9

A quantitative and site-specific atlas of the citrullinome reveals widespread existence of citrullination and insights into PADI4 substrates

In the format provided by the authors and unedited

Supplementary notes to “A quantitative and site-specific atlas of the citrullinome reveals widespread existence of citrullination and insights into PADI4 substrates” by Rebak et al.

Supplementary note 1

The elution times of citrullinated peptides were compared to their individual isotope counterparts containing ^{13}C and ^{15}N isotopes. Given that our analytical setup encompasses two sequential separation stages involving an initial offline high pH liquid chromatography (LC) step, followed by a subsequent online low pH LC-MS stage, we investigated the elution of all peptides in both stages. During the high pH LC separation, we achieved a distinct separation between citrullinated peptides and their unmodified counterparts, with citrullinated peptides consistently eluting earlier than their unmodified counterparts, as depicted in Fig. 2E. Additionally, the non-citrullinated peptides elute earlier during the low pH LC separation (Fig.S2E). Consequently, citrullinated peptides and unmodified counterparts seldom coexist in the same MS full scan, significantly diminishing the potential for their co-fragmentation and consequently reducing the likelihood of erroneously identifying ^{13}C peaks as citrullination events. We found that deamidated peptides included in the non-citrullinated peptides differ in charge state compared to citrullinated peptides (Fig.S2F). Thus, the mass-to-charge (*m/z)* state of citrullinated peptides remains overall higher than that of deamidated peptides, hereby limiting co-fragmentation of citrullinated and deamidated peptides. To further this, we conducted an additional examination of the spatial proximity between the identified citrullination sites and the nearest asparagine (N) and glutamine (Q) residues. This investigation aimed to assess whether the citrullination identification process introduced any bias that could lead to the misclassification of nearby N and Q residues as citrullination events. In summary, our findings indicate the absence of any spatial bias, thus confirming that our analytical separation approach coupled with high-resolution mass spectrometry and confidence scoring enables the precise localization of citrullination sites with high confidence (Fig.S2G).

Supplementary note 2

We evaluated the inhibitory concentration needed to reduce the total cellular levels of citrullination by 50% (Fig.3E, light blue line). It is important to acknowledge that citrullination regulation occurs at the site level for each substrate, implying that proteins may harbor modification sites with different citrullination kinetics. To take this into account, we additionally determined when the cumulative signal of all citrullination sites would be reduced in their expression levels by 50% (Fig.3E, dark blue line). Combining both approximations yielded a cellular GSK484 concentration value of 1-3µM, which is roughly 20 times higher than the IC50 reported for GSK484. This difference likely reflects that our analysis was performed in living cells and in the presence of calcium, which is known to reduce the

inhibitory effect of PADI4 inhibitors[1]. Moreover, our calculations were based upon abundance levels related to the entire proteomics-derived citrullinome, whereas standard IC50 values often are based upon low-throughput analysis of a few substrates[2].

Supplementary note 3

While histone citrullination is subject to widespread regulation, it is worth noting that specific markers, like H3R8, exhibited a comparatively modest reduction following GSK484 treatment. This suggests the possibility that these sites may be influenced by other PADIs, or that their turn-over is slower when compared to other histone marks such as H3R17 and H3R26. In fact, PADI2 is reported to target the same histone marks as PADI4 and while we only observe low cellular expression of PADI2 the enzyme may still be able to catalyze low-level citrullination events. Still, for H3R8 we observed a significant regulation in citrullination upon GSK484 (Table S3), which suggests that PADI4 is the major enzyme responsible for the catalysis of observed histone citrullination sites. In general, our data support that PADI4 has the capability to hypercitrullinate histones[3,4] and to a larger degree than previously recognized.

Supplementary note 4

While considering citrullination of transcriptional regulators, we next investigated if the DNA binding regions were significantly targeted. Across all citrullination sites detected in our screen, we observed a significant preference for targeting DNA binding regions, when compared to a background comprising all arginine residues within the target proteins (Fig.S4A). This preferential targeting of DNA binding regions was also observed just within the 179 transcriptional regulators citrullinated by PADI4, when compared to a background comprising all arginine residues within the 179 proteins (Fig.S4B). These finding support that PADI4-mediated citrullination might have a functional role in the regulation of transcriptional regulators, potentially modulating their ability to interact with DNA.

Supplementary note 5

To explore which biological processes are affected by the observed citrullination events, we decided to focus on the downstream targets of the citrullinated transcription factors and investigate which pathways are enriched. While this analysis provides an approximate assessment, we found a high enrichment for pathways related to antimicrobial response and killing of foreign cells, as well as keratinization and cornification (Fig.S4C). These pathways are known to be linked to citrullination[5,6], albeit not at a gene expression level, which provides another level of complexity in understanding the role of citrullination in for example NETosis and myelination[7,8]. Taken together, our results offer a systems-wide overview of PADI4-regulated citrullination sites spanning both histone and non-

histone proteins. These results can serve as a valuable resource for generating hypotheses regarding the potential biological implications of citrullination.

Supplementary note 6

The cells were pelleted by centrifugation and the Lockes buffer was removed. Cell pellets were then lysed in 10 pellet volumes of Lysis Buffer (6M guanidine-HCl, 50 mM TRIS, pH 8.5). Rapid cell lysis was achieved by alternating vigorous vortexing and vigorous shaking of the samples, 5 seconds per cycle and for 30 seconds in total, after which the lysates were snap frozen in liquid nitrogen. The lysates were stored at −80°C until further sample processing. Lysates were thawed at room temperature (RT) and homogenized using a microtip sonicator, via two pulses of 10 seconds at 30 W. The homogenized lysates were reduced and alkylated, by the addition of Tris(2-carboxyethyl)phosphine (TCEP) and chloroacetamide (CAA), both to a final concentration of 5mM, and incubation for one hour at RT . Proteins were digested using Lysyl Endopeptidase (Lys-C, 1:100 w/w) (cat. 129-02541, Wako Chemicals) for 3 hours at RT. Following a three-fold dilution with 50mM Tris, a second round of Lys-C (1:200 w/w) digestion was performed overnight at RT. Following digestion, samples were acidified via addition of trifluoroacetic acid (TFA) to a final concentration of 0.5% (v/v).

Supplementary note 7

Peptides were loaded onto the column at 1 mL/min for 4 min, after which they were separated on a linear gradient ranging from 5%B to 25%B over 62 min. followed by an increase to 60 %B over 5 min and 70%B over 3min. Fractions were collected into a 96-deep well plate every 60 to 90 seconds. Following the primary gradient, fraction collection was stopped and the column flow was kept at 70%B for an additional 5 min before it was reduced to 5%B. Buffer C was constant throughout the gradient at 10%.

Supplementary note 8

Default MaxQuant setting were used with exceptions outlined below.. Enzyme cleavage specificity was set to Lys-C. Protein N-terminal acetylation, oxidation (M), phosphorylation (S, T, and Y), deamidation (N, Q, and R), methylation (K and R), di-methylation (K and R), tri-methylation (K), and acetylation (K), were all included as variable modifications. Citrullination, i.e. deamidation of R, was further defined with expected neutral loss of cyanic acid (HNOC, 43.01 Da), and with the immonium ion as diagnostic peak (H11C5N3O, 129.09 Da). A maximum of 3 variable modifications per peptide

and a maximum of 2 missed cleavages were allowed. Matching between runs was enabled, with a match window of 0.7 minutes and an alignment time window of 20min. Default settings for filtering by posterior error probability were used to achieve a false discovery rate of <1% at the peptide-spectrum match, protein assignment, and site-specific levels. Label free quantification and iBAQ were enabled.

Supplementary note 9

For statistical analysis of the microarray data, we took the top 2,000 most enriched peptide pairs in synovial fluid from CCP positive patients, selected by the highest z-scores, and performed Fisher Exact testing versus several groups of citrullination sites. The combined list of all (24,505) peptide pairs in the experiment, as derived from all groups and arrays, was used as a background. The tested foreground groups were; top 250, top 500, top 1000, top 2000, top 3000, top 5000, or all sites identified in our mass spectrometry screen ranked by overall abundance (intensity), as well as a group of (2,788) peptides bearing multiple citrullines co-modifying the same peptide sequences as identified in our screen. Additionally; a random sampling of 250, 500, 1000, 2000, 3000, 5000, or all peptides from the randomly generated citrullinated peptides printed on the array. Finally; we included the linearized variant of the PEPperPRINT commercial citrulline library (334 pairs). False discovery rate was controlled via application of Benjamini-Hochberg correction for multiple hypothesis testing. For visualization, we calculate a score based on the logarithm of the enrichment ratio multiplied by the negative logarithm of the q-value, which we then scaled into a relative percentage score for ease of interpretation.

Bibliography for supplementary notes

1. Lewis, H.D. et al. Inhibition of PAD4 activity is sufficient to disrupt mouse and human NET formation *Nat Chem Biol* **11**, 189-191 (2015).
2. Mondal, S. & Thompson, P.R. Protein Arginine Deiminases (PADs): Biochemistry and Chemical Biology of Protein Citrullination. *Accounts of Chemical Research* **52**, 818-832 (2019).
3. Leshner, M. et al. PAD4 mediated histone hypercitrullination induces heterochromatin decondensation and chromatin unfolding to form neutrophil extracellular trap-like structures. *Frontiers in Immunology* **3**, 1-11 (2012).
4. Wang, Y. et al. Histone hypercitrullination mediates chromatin decondensation and neutrophil extracellular trap formation. *J. Cell Biol* **184**, 205-213 (2009).
5. Ishida-Yamamoto, A. et al. Sequential reorganization of cornified cell keratin filaments involving filaggrin-mediated compaction and keratin 1 deimination. *Journal of Investigative Dermatology* **118**, 282-287 (2002).
6. Méchin, M.C., Takahara, H. & Simon, M. Deimination and peptidylarginine deiminases in skin physiology and diseases. *International Journal of Molecular Sciences* **21**, 1-15 (2020).

7. Corsiero, E., Pratesi, F., Prediletto, E., Bombardieri, M. & Migliorini, P. NETosis as source of autoantigens in rheumatoid arthritis. *Frontiers in Immunology* **7**, 1-9 (2016).
8. Falcão, A.M. et al. PAD2-Mediated Citrullination Contributes to Efficient Oligodendrocyte Differentiation and Myelination. *Cell Reports* **27**, 1090-1102.e10 (2019).
