# Supplementary material for: A quantitative and site-specific atlas of the citrullinome reveals widespread existence of citrullination and insights into PADI4 substrates
Source: Nat Struct Mol Biol. 2024 Feb 6;31(6):977–95. doi: 10.1038/s41594-024-01214-9 (PMC11189309; doi:10.1038/s41594-024-01214-9)
Supplement: Supplementary file 2 — Reporting Summary [file 41594_2024_1214_MOESM2_ESM.pdf]

Corresponding author(s): Michael L. Nielsen

Last updated by author(s): June 27 2023

Reporting Summary

Nature Portfolio wishes to improve the reproducibility of the work that we publish. This form provides structure for consistency and transparency in reporting. For further information on Nature Portfolio policies, see our Editorial Policies and the Editorial Policy Checklist.

Statistics

For all statistical analyses, confirm that the following items are present in the figure legend, table legend, main text, or Methods section.

n/a	Confirmed	
☐	☒	The exact sample size (*n*) for each experimental group/condition, given as a discrete number and unit of measurement
☐	☒	A statement on whether measurements were taken from distinct samples or whether the same sample was measured repeatedly
☐	☒	The statistical test(s) used AND whether they are one- or two-sided<br>*Only common tests should be described solely by name; describe more complex techniques in the Methods section.*
☒	☐	A description of all covariates tested
☐	☒	A description of any assumptions or corrections, such as tests of normality and adjustment for multiple comparisons
☐	☒	A full description of the statistical parameters including central tendency (e.g. means) or other basic estimates (e.g. regression coefficient) AND variation (e.g. standard deviation) or associated estimates of uncertainty (e.g. confidence intervals)
☐	☒	For null hypothesis testing, the test statistic (e.g. F, t, r) with confidence intervals, effect sizes, degrees of freedom and P value noted<br>*Give P values as exact values whenever suitable.*
☒	☐	For Bayesian analysis, information on the choice of priors and Markov chain Monte Carlo settings
☒	☐	For hierarchical and complex designs, identification of the appropriate level for tests and full reporting of outcomes
☐	☒	Estimates of effect sizes (e.g. Cohen's *d*, Pearson's *r*), indicating how they were calculated

Our web collection on statistics for biologists contains articles on many of the points above.

Software and code

Policy information about availability of computer code

Data collection	No custom software was used. The MS data was collected with the software provided with the instruments.
Data analysis	No custom software was used. All software used in the manuscript is publicly available and described in the methods. Software used: MaxQuant v1.5.3.30, Perseus v1.6.14.0, iceLogo v1.2, IceLogo v1.3.8, BoxPlorR web tool v1 and Sicasys v1.4 were used.

For manuscripts utilizing custom algorithms or software that are central to the research but not yet described in published literature, software must be made available to editors and reviewers. We strongly encourage code deposition in a community repository (e.g. GitHub). See the Nature Portfolio guidelines for submitting code & software for further information.

Data

Policy information about availability of data

All manuscripts must include a data availability statement. This statement should provide the following information, where applicable:

- Accession codes, unique identifiers, or web links for publicly available datasets
- A description of any restrictions on data availability
- For clinical datasets or third party data, please ensure that the statement adheres to our policy

The mass spectrometry proteomics data have been deposited to the ProteomeXchange Consortium via the PRIDE (Perez-Rivero et al., 2022) partner repository with the dataset identifier PXD038702
All other data generated in this study is provided in the Supplementary Information/Source Data file. Source data are provided with this paper.

Databases used in no particular order: AlphaFold116, UniProtKB104, DAVID Bioinformatics94, AAgAtlas 1.045, ReMap2020100 and GeneHancer Double Elite101.

Research involving human participants, their data, or biological material

Policy information about studies with human participants or human data. See also policy information about sex, gender (identity/presentation), and sexual orientation and race, ethnicity and racism.

Reporting on sex and gender	Sex and gender was not considered in the study design, instead patients were recruited based on needing joint-puncture and steroid treatment due to flare in the joint.<br>A total of 18 patients were included, 12 females and 6 males. Patient ages at point of sample collection were 28, 29, 29, 31, 31, 33, 44, 49, 51, 54, 61, 62, 62, 63, 67, 72, 73, 76 years old.
Reporting on race, ethnicity, or other socially relevant groupings	There were no relevant race, ethnicity or other socially relevant categorization variable(s) used in the manuscript.
Population characteristics	The patients were divided into three groups based on diagnosis, Anti-CCP-negative rheumatoid arthritis patients, Anti-CCP-positive rheumatoid arthritis patients and patients suffering from ankylosing spondylitis. Ages ranged from 28 to 76 years old at point of sample collection.
Recruitment	Patients with RA and SpA with a clinical single joint flare where the treating physician found indication for joint puncture and aspiration - with or without glucocorticoid injection - were recruited for the project and referred for an ultrasound guided procedure by a rheumatologist related to the project thereby minimizing the self-selection bias. Patients with recent trauma to or surgery of the involved joint or who had had a glucocorticoid injection in the same joint within 3 months were excluded. None of the recruited patients declined participation in the project. We only included patients with a single joint flare and hence the inflammation level may be higher in patients with generalized flare. These patients would however never be treated with joint aspiration and injections alone. Patients were not compensated for participation.
Ethics oversight	Synovial fluid was from collected patients suffering from anti-CCP-positive RA, patients with anti-CCP-negative RA, and patients with ankylosing spondylitis, all recruited at Center for Rheumatology and Spine Diseases, Copenhagen University Hospital Glostrup after informed consent after approval by the local ethical committee (H-16042831). The local ethics committee was: De Videnskabsetiske Komiteer for Region Hovedstaden, Regionsgården Kongens Vænge 2, 3400 Hillerød, Denmark with journal nr.:H-16042831.

Note that full information on the approval of the study protocol must also be provided in the manuscript.

Field-specific reporting

Please select the one below that is the best fit for your research. If you are not sure, read the appropriate sections before making your selection.

☒ Life sciences ☐ Behavioural & social sciences ☐ Ecological, evolutionary & environmental sciences

For a reference copy of the document with all sections, see nature.com/documents/nr-reporting-summary-flat.pdf

Life sciences study design

All studies must disclose on these points even when the disclosure is negative.

Sample size	No sample-size calculation was performed. MS analysis were performed in biological triplicates or quadruplicates to ensure reproducibility using HL60 cell line lysates. The size of individual samples (protein starting material) was determined during pilot experiments.<br>The peptide microarray analysis was performed as a single replicate, n=1, due to the expansive nature of this analysis.
Data exclusions	One replicates for all conditions was excluded in study 1 of the MS analysis, due to unfortunate sample loss prior to MS analysis and this study is therefore performed in triplicates.
Replication	MS experiments were perfomed in triplicate or quadruplicates to ensure reproducibility as shown by principal component analysis (figure S1A) and pearson correlation (S1B). All other MS experiments similarly showed reproducibility.<br>WB analysis was performed in at least duplicates. All attempts of replication were successful.<br>The peptide microarray analysis was performed as a single replicate, n=1, due to this no statistical comparison between the microarrays was performed only within the same array.
Randomization	Samples were not divided into experimental groups.<br>All replicates for all individual mass spectrometry experiments were prepared simultaneously and statistically processed while taking multiple-hypothesis testing into account.
Blinding	Sample preparation was not blinded, all samples relating to each experiment were prepared simultaneously and clearly labeled, which is important for MS analysis.<br>During MS data acquisition, the performance of the MS instrument drifts over time or power outages and other factors outside our control may affect acquisition. Samples were therefore run in an order that introduces the least technical variance.

All MS data is publicly available for reprocessing or investigation by any external party.
Western blot analysis was also not blinded due to the importance of keeping track of samples.

Reporting for specific materials, systems and methods

We require information from authors about some types of materials, experimental systems and methods used in many studies. Here, indicate whether each material, system or method listed is relevant to your study. If you are not sure if a list item applies to your research, read the appropriate section before selecting a response.

Materials & experimental systems

n/a	Involved in the study
☐	☒ Antibodies
☐	☒ Eukaryotic cell lines
☒	☐ Palaeontology and archaeology
☒	☐ Animals and other organisms
☒	☐ Clinical data
☒	☐ Dual use research of concern
☒	☐ Plants

Methods

n/a	Involved in the study
☒	☐ ChIP-seq
☒	☐ Flow cytometry
☒	☐ MRI-based neuroimaging

Antibodies

Antibodies used

The following antibodies were used in this study: rabbit polyclonal PADI4 (cat. P4749, Sigma Aldrich), rabbit monoclonal H3 (citrulline Arg2) antibody (1:1000, cat. Ab176843, clone EPR17703, Abcam) and rabbit polyclonal GAPDH (1:1000, Ab9485, Abcam). The Anti-Citrulline (Modified) Detection Kit (cat. 17-347B, Merck) was used to measure global citrullination34, according to the manufacturer's instructions.
Global citrullination was detected with the antimodified citrulline (AMC) Detection Kit (cat. 17-347B, Merck) according to the manufacturer's instructions.
Goat anti-human IgG (Fc) DyLight680 was used for testing secondary binding of microarrays (0.1 µg/ml, cat. SA5-10138, Thermo Fisher Scientific)

Validation

Anti-PADI4 (P4749) is according to Sigma Aldrich validated for WB. H3Cit (Ab176843) is validated by Abcam for WB, the same applied for anti-GAPDH (Ab9485). Thermo Fisher Scientific states that the Goat anti-human IgG (SA5-10138) is validated for immunoassays.

Eukaryotic cell lines

Policy information about cell lines and Sex and Gender in Research

Cell line source(s)

HL60 cell line derived from peripheral blood lymphocytes of a women suffering from acute promyleocytic leukaemia. The HL60 cell line was gifted to us by Maria Christophorou.

Authentication

The cell line was not authenticated

Mycoplasma contamination

The cell lines were not tested for mycoplasma contamination

Commonly misidentified lines
(See ICLAC register)

No commonly misidentified cell lines were used in this study.
