# Supplementary material for: A quantitative and site-specific atlas of the citrullinome reveals widespread existence of citrullination and insights into PADI4 substrates
Source: Nat Struct Mol Biol. 2024 Feb 6;31(6):977–95. doi: 10.1038/s41594-024-01214-9 (PMC11189309; doi:10.1038/s41594-024-01214-9)

Figure 1B, Western blotting PADI4, H3Cit and GAPDH
weight marker is Invitrogen SeeBlue Plus2 Pre-stained Protein Standard

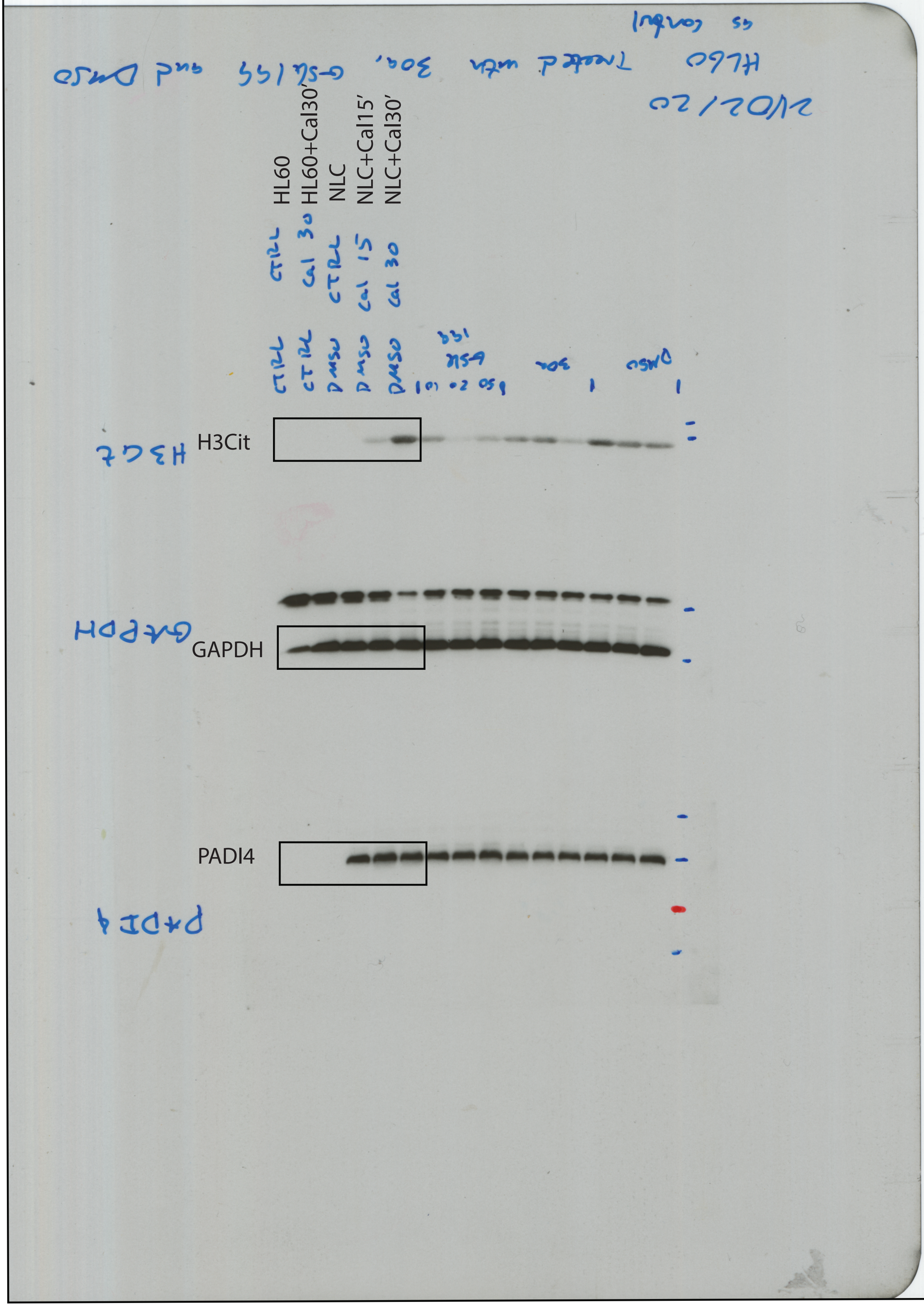

Figure 1B, Western blotting ModCit
See next page for scan of longer exposure with weight marker
(Invitrogen SeeBlue Plus2 Pre-stained Protein Standard)

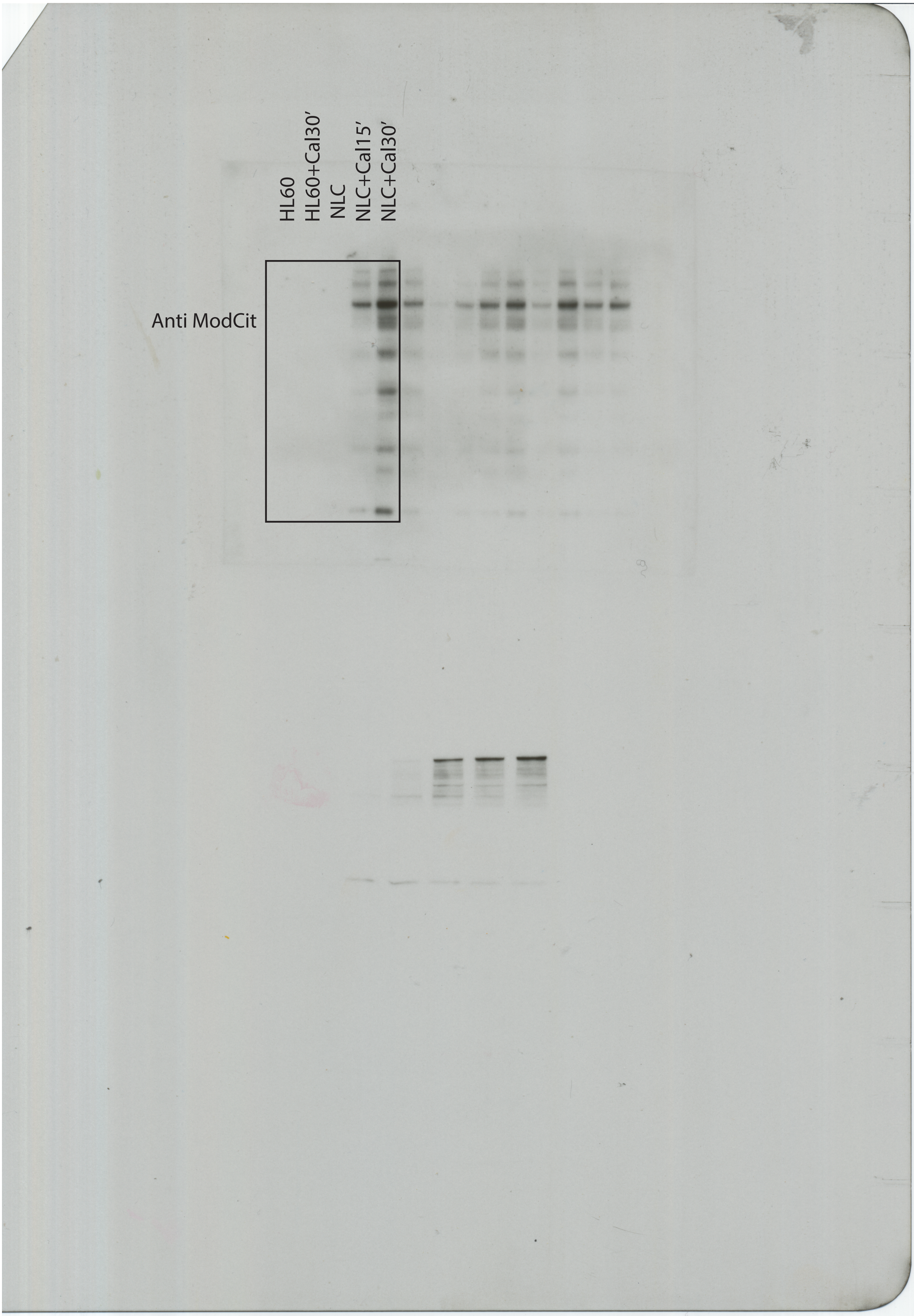

Figure 1B, Western blotting ModCit
(Scan of longer exposure with weight marker (Invitrogen SeeBlue Plus2 Pre-stained Protein Standard)

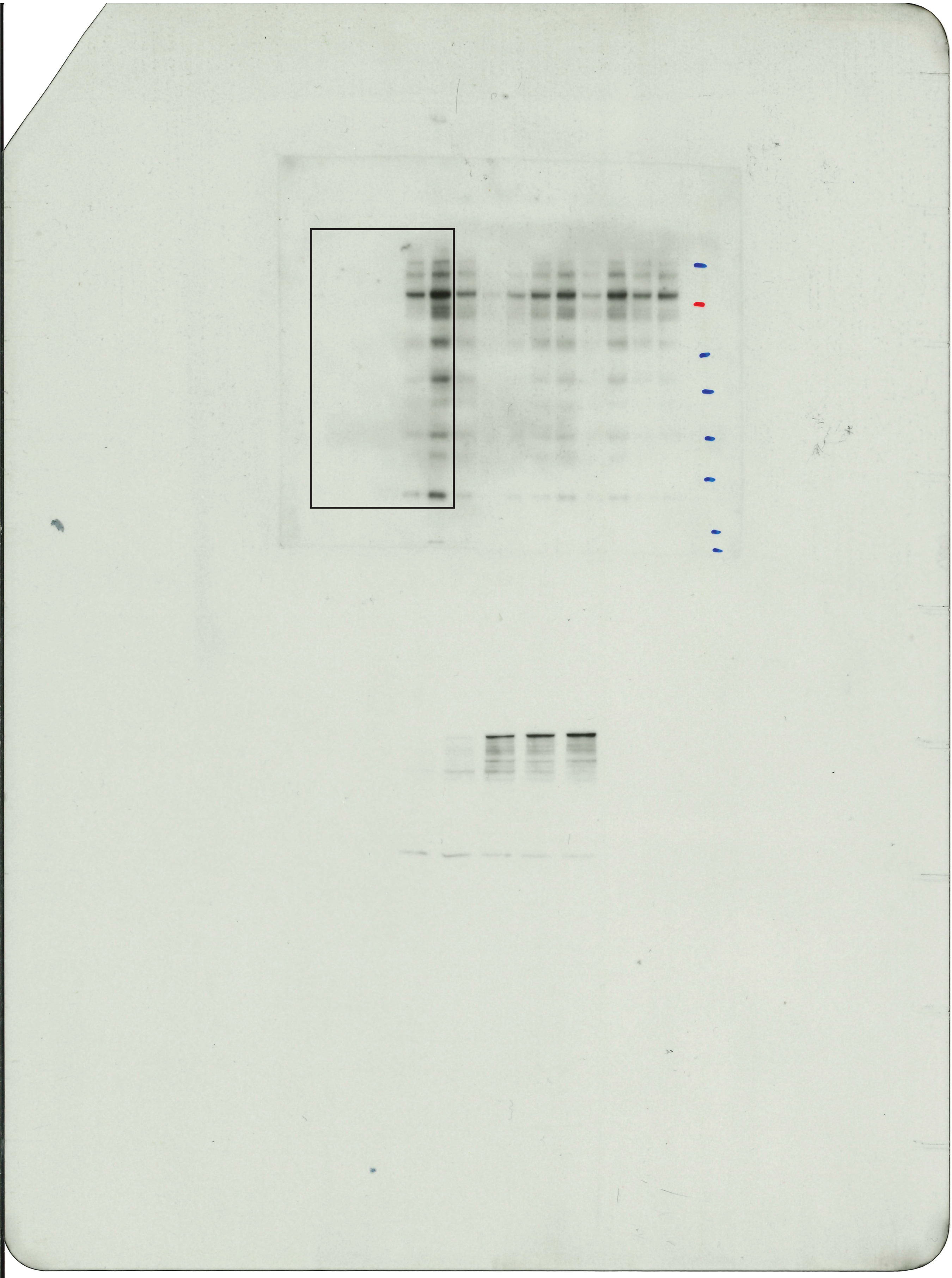

Supplement: Supplementary file 4 — Unprocessed western blots for Fig. 1b. [file 41594_2024_1214_MOESM4_ESM.pdf]
