# Supplementary material for: A quantitative and site-specific atlas of the citrullinome reveals widespread existence of citrullination and insights into PADI4 substrates
Source: Nat Struct Mol Biol. 2024 Feb 6;31(6):977–95. doi: 10.1038/s41594-024-01214-9 (PMC11189309; doi:10.1038/s41594-024-01214-9)

Figure 3B - Western blotting H3Cit
weight marker is Invitrogen SeeBlue Plus2 Pre-stained Protein Standard

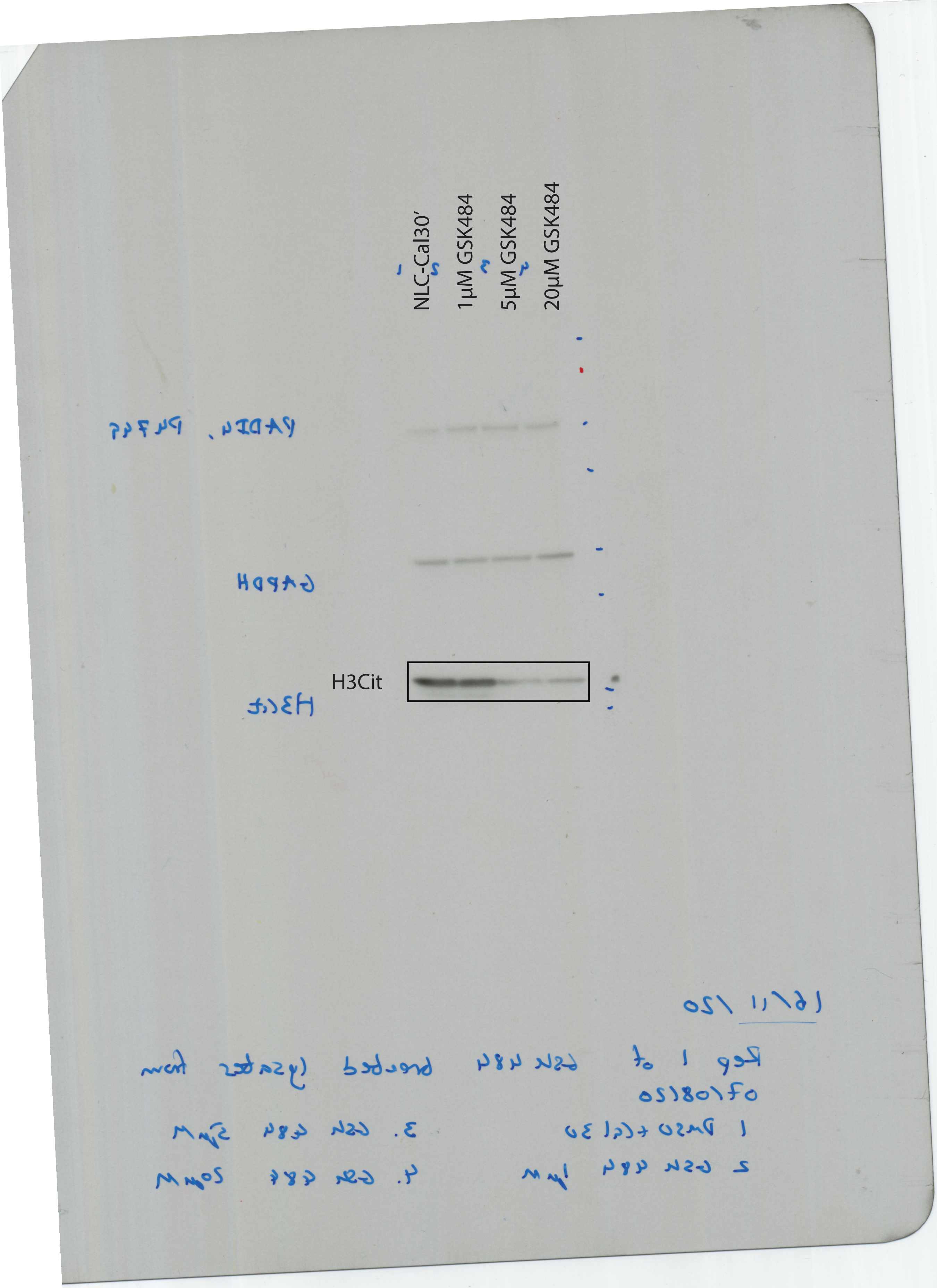

Figure 3B - Western blotting PADI4, GAPDH
weight marker is seen on previous page with shorter exposure
(Invitrogen SeeBlue Plus2 Pre-stained Protein Standard)

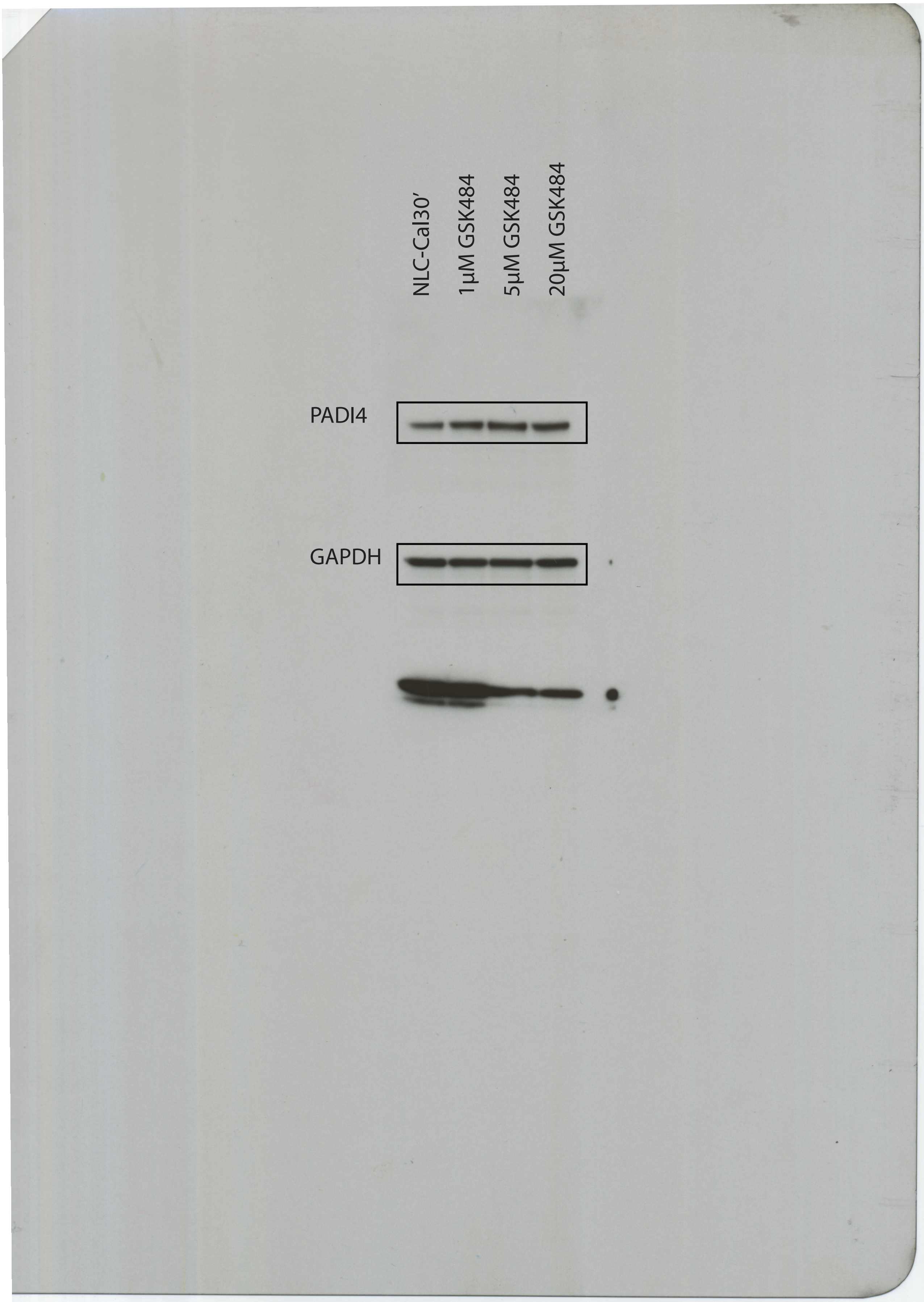

Figure 3B - Western blotting ModCit
weight marker is Invitrogen SeeBlue Plus2 Pre-stained Protein Standard

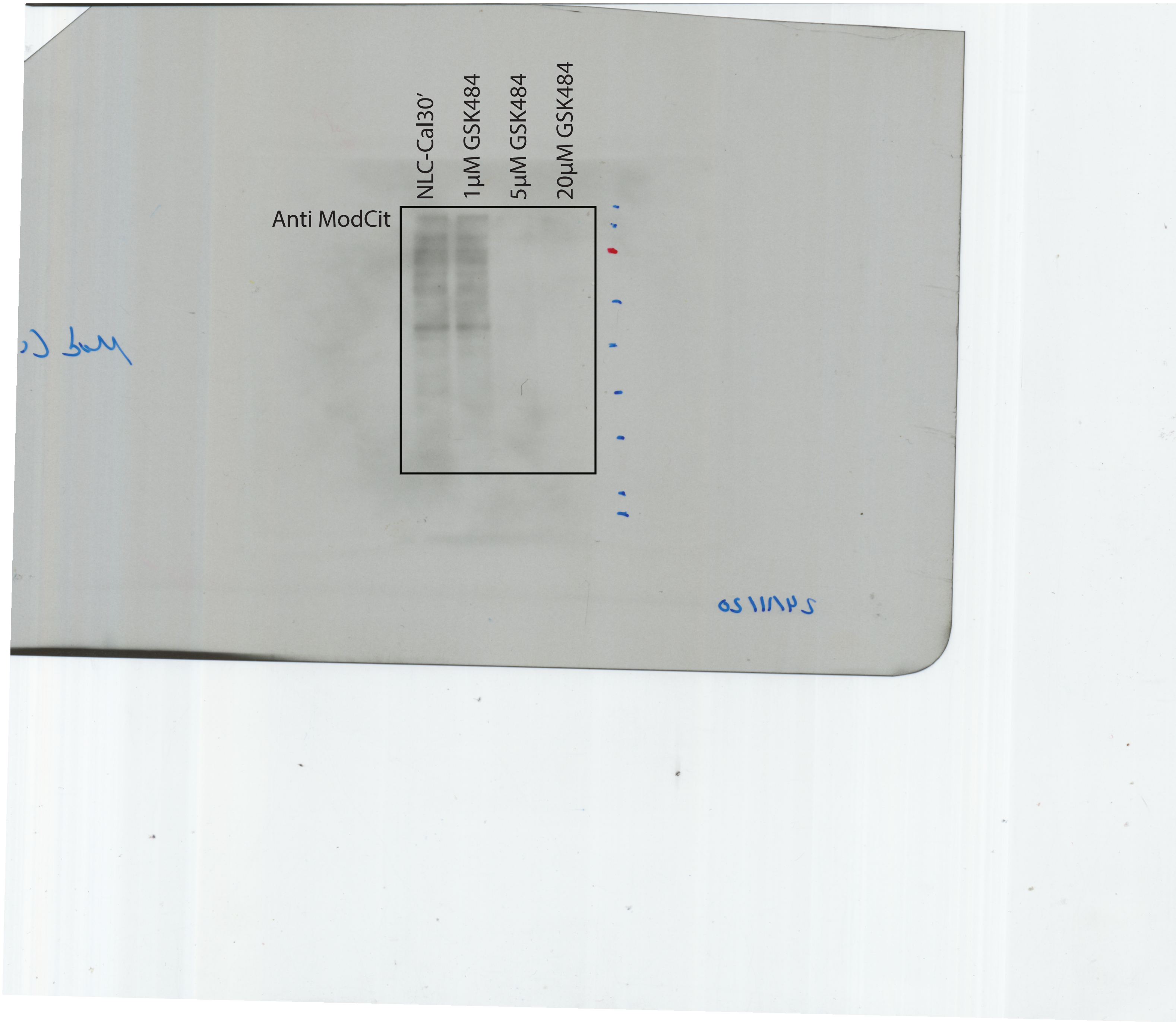

Supplement: Supplementary file 5 — Statistical source data for Fig. 1. [file 41594_2024_1214_MOESM5_ESM.pdf]
